# An Intrinsically Conductive Cross‐Conjugated Polymer with a Quinhydrone‐Like Donor–Acceptor Charge‐Transfer Network

**DOI:** 10.1002/anie.202518109

**Published:** 2025-11-04

**Authors:** Naixin Zhao, Yonglin Wang, Xin Jin, Yuning Li

**Affiliations:** ^1^ Department of Chemical Engineering Waterloo Institute for Nanotechnology (WIN) University of Waterloo 200 University Ave West Waterloo Ontario N2L 3G1 Canada

**Keywords:** Charge transfer complex, Cross‐conjugated polymers, Intrinsic doping, Polaron, Quinhydrone

## Abstract

Intrinsically conductive polymers free of unstable mobile dopants are highly sought after for stable and reliable electronic performance, yet remain scarce due to synthetic challenges. Here we report poly(3,4‐dihydroxythiophene‐*alt*‐thiophene‐3,4‐dione) (HOT‐DOT), a novel cross‐conjugated polymer and the first polymeric analogue of a quinhydrone‐like charge‐transfer complex with intrinsic conductivity. HOT‐DOT is synthesized through a straightforward three‐step route, in which the final air oxidation step spontaneously generates a perfectly balanced 1:1 donor–acceptor architecture that promotes self‐doping and stabilizes polarons. The polymer exhibits a narrow bandgap (1.38 eV), broad near‐infrared absorption, and high conductivity (∼0.29 S cm^−1^), enabled by an ultrasmall π–π stacking distance (3.25 Å) despite its cross‐conjugated backbone. Spectroscopic and computational analyses reveal that strong interchain donor–acceptor interactions, reinforced by ammonia coordination, stabilize the self‐doped state. HOT‐DOT further displays rare positive temperature coefficient (PTC) behavior and long‐term ambient stability. As a proof of concept, flexible temperature sensors fabricated from HOT‐DOT films show reproducible and linear thermal responses over multiple cycles. This study establishes polymeric charge‐transfer complexes as a new design paradigm for intrinsically conductive, dopant‐free polymers with distinctive transport and sensing properties.

## Graphical abstract

HOT‐DOT, an intrinsically conductive cross‐conjugated polymer, features a covalently integrated, quinhydrone‐like donor–acceptor network formed through spontaneous air oxidation. This built‐in architecture enables self‐doping, stable polarons, and efficient charge transport without dopants. HOT‐DOT films show high conductivity and rare positive temperature coefficient, offering a new paradigm for organic electronic materials.

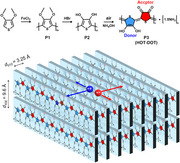

## Introduction

Conductive polymers have revolutionized electronics, offering mechanical flexibility, tunable optoelectronic properties, and compatibility with solution processing for applications ranging from displays and sensors to energy storage.^[^ ^1^, ^2^, ^3^, ^4^, ^5^, ^6^, ^7^, ^8^, ^9^ ^]^ Yet, a persistent challenge remains: most π‐conjugated polymers are electrically insulating in their pristine state and require chemical doping with mobile counterions to achieve conductivity.^[^ ^10^, ^11^, ^12^, ^13^ ^]^ Such dopants introduce instability, moisture sensitivity, and long‐term degradation, severely limiting practical applications.

To overcome these limitations, intrinsically conductive polymers that generate charge carriers without external dopants have been pursued.^[^ ^14^ ^]^ Common strategies include incorporating dopant‐like side chains, such as sulfonic acid groups^[^ ^14^, ^15^, ^16^ ^]^ or embedding stable radical moieties that act as intrinsic charge carriers.^[^ ^17^, ^18^ ^]^ For example, certain donor–acceptor (D–A) conjugated polymers can adopt open‐shell diradical resonance forms, enabling high conductivity (> 10^−1^ S cm^−1^) in the pristine state,^[^ ^19^ ^]^ which far exceeds that of conventional closed‐shell polymers (e.g., undoped poly(3‐hexylthiophene): ∼10^−9^ S cm^−1^).^[^ ^17^ ^]^ This high conductivity is attributed to the stabilization of the open‐shell configuration through extended conjugation and delocalization of unpaired electrons, which facilitates efficient long‐range charge transport.^[^ ^19^ ^]^ Alternatively, non‐conjugated radical polymers bearing stable radical sites on each repeat unit can conduct via redox‐hopping mechanisms,^[^ ^18^, ^20^, ^21^ ^]^ achieving conductivities on the order of 10^−1^ S cm^−1^ at elevated temperatures (e.g., 0.28 S cm^−1^ at 80 °C).^[^ ^20^ ^]^ These examples demonstrate that intrinsic charge generation can serve as a viable substitute for traditional doping strategies.

A promising yet underexplored strategy for achieving self‐doping is the use of polymeric charge‐transfer complexes (CTCs). In classical small‐molecule CTCs such as tetrathiafulvalene–tetracyanoquinodimethane (TTF–TCNQ), electron‐rich donor molecules transfer charge (electron) to electron‐deficient acceptors, generating radical cations and anions (i.e., polarons) that serve as mobile charge carriers within stacked π‐networks.^[^ ^22^, ^23^ ^]^ These systems can exhibit metallic‐level conductivities (∼10^3^ S cm^−1^), but their practical application is hindered by poor processability, mechanical fragility, and the need for precise stoichiometry. In contrast, polymeric analogues, in which donor (D) and acceptor (A) units are covalently linked in a fixed 1:1 ratio along the backbone, offer improved structural stability, ease of processing, and built‐in control over D–A interactions. Such systems could, in principle, enable intrinsic charge generation via both intrachain and interchain charge transfer, eliminating the need for external dopants. However, despite their conceptual appeal, well‐defined polymeric CTCs with covalently integrated, stoichiometric donor–acceptor architectures have yet to be realized, largely due to synthetic challenges and the limited availability of suitable monomers.

**Figure 1:**
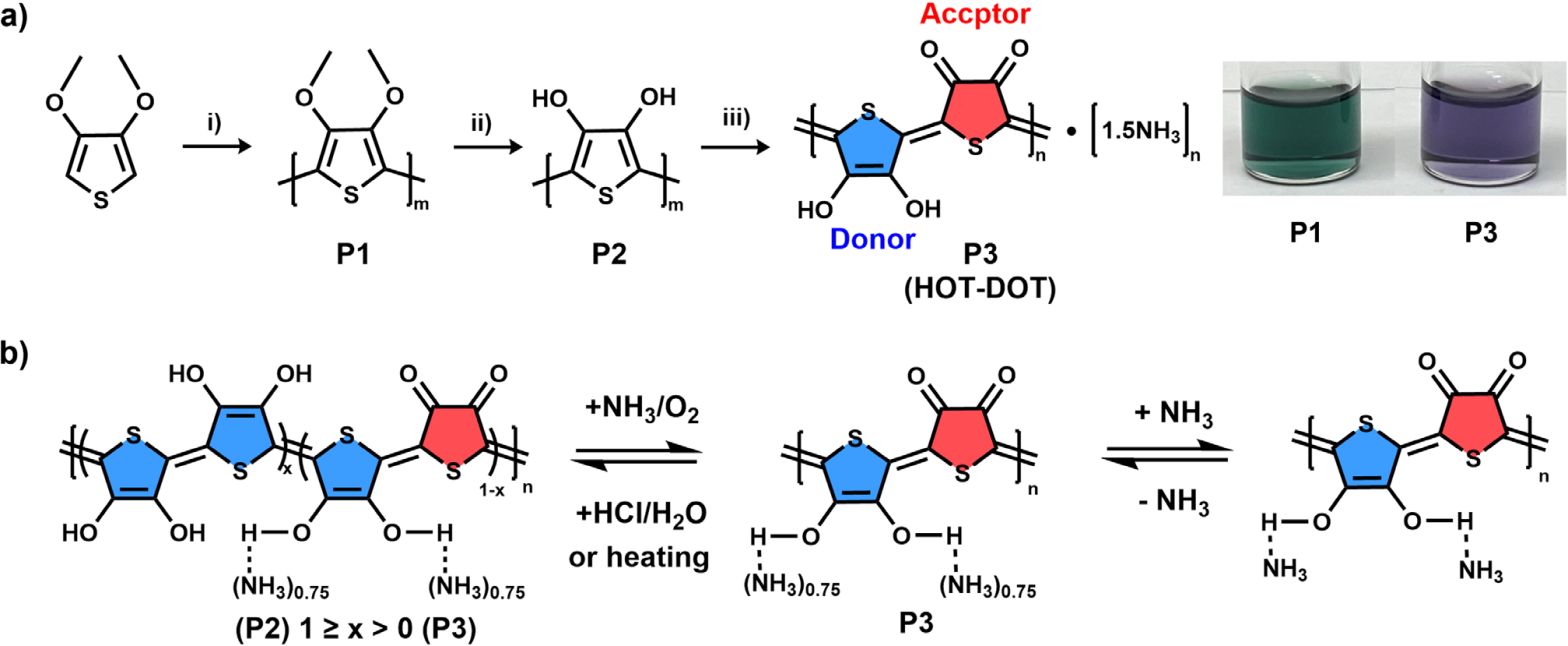
a) Synthetic route to P3 (HOT‐DOT), an intrinsically conductive and solution‐processable polymer composed of 50% electron‐rich dHOT units and 50% electron‐deficient dOT units, as reported in this work. Reaction conditions: (i) FeCl_3_, 0 °C, 48 h; (ii) 48 wt% HBr in acetic acid, 120 °C, 12 h (yield: 88.6%); (iii) 33 wt% aqueous NH_4_OH, room temperature, air, 30 min (quantitative yield). b) Reversible structural transformations of P3 upon exposure to ammonia, HCl, or thermal treatment.

In this work, we present poly(3,4‐dihydroxythiophene‐*alt*‐thiophene‐3,4‐dione) (HOT‐DOT, P3; Figure 1), a thiophene‐based cross‐conjugated polymer that represents the first polymeric analogue of a quinhydrone‐like donor–acceptor charge‐transfer complex. P3 is synthesized through a simple three‐step sequence: i) oxidative polymerization of 3,4‐dimethoxythiophene to yield poly(3,4‐dimethoxythiophene) (P1); ii) demethylation to form poly(3,4‐dihydroxythiophene) (P2); and iii) a spontaneous, self‐limiting oxidation of P2 in aqueous ammonia under ambient air, which automatically converts half of the dihydroxythiophene (dHOT) units into electron‐deficient thiophene‐3,4‐dione (dOT) units while ammonia coordinates with the remaining dHOT sites. This unique reaction yields a perfectly balanced 1:1 D–A ratio and a covalently bonded, alternating donor–acceptor architecture without the need for external stoichiometric control, closely resembling the classical air‐oxidation of hydroquinone to form hydroquinone–benzoquinone (quinhydrone) complexes.^[^ ^24^, ^25^ ^]^

P3 exhibits a narrow optical bandgap (∼1.38 eV) and strong near‐infrared absorption extending beyond 1200 nm, characteristic of delocalized radical ions (polarons). Red‐shifted and intensified absorption in the solid state suggests significant interchain charge‐transfer interactions. Electron paramagnetic resonance (EPR) spectroscopy confirms the presence of unpaired electrons, with a radical concentration of ∼1.24 mol% relative to the thiophene units, supporting the formation of polarons via internal donor–acceptor charge transfer. Notably, P3 achieves a conductivity of ∼0.29 S cm^−1^ in the absence of any external dopant, rivalling the performance of leading intrinsically conductive polymers.

Despite its cross‐conjugated backbone, which typically hinders efficient charge transport,^[^ ^26^ ^]^ P3 exhibits a respectable carrier mobility (∼0.028 cm^2^ V^−1^ s^−1^). This performance is attributed to effective charge transport through densely packed D–A π–π stacks, as supported by X‐ray diffraction (XRD) analysis, which reveals an exceptionally short π–π stacking distance of 3.25 Å. Such close packing facilitates charge hopping and enhances mobility. P3 also displays a positive temperature coefficient (PTC) of resistance, an uncommon feature among organic semiconductors,^[^ ^27^ ^]^ likely resulting from thermally induced disruptions in π–π stacking that impede charge transport. Importantly, P3's electrical properties remain stable over at least two months under ambient conditions, highlighting the advantage of its dopant‐free design and the absence of mobile dopant species.

To demonstrate its practical utility, flexible temperature sensors were fabricated using solution‐processed thin films of P3. The devices exhibit a reproducible and linear resistance response over the 25–45 °C range, with a temperature coefficient of resistance (TCR) of 0.113% °C^−1^. They maintained stable performance across multiple thermal cycles and after extended ambient storage, underscoring the potential of dopant‐free CTC polymers like P3 for robust, flexible electronic applications.

## Results and Discussion

### Material Synthesis

P3 was synthesized following the route outlined in Figure 1a, with experimental details provided in the Supporting Information. First, poly(3,4‐dimethoxythiophene) (P1) was prepared via oxidative coupling polymerization of 3,4‐dimethoxythiophene using iron(III) chloride (FeCl_3_) at 0 °C for 48 h under a nitrogen atmosphere. The crude product was precipitated in methanol and extensively washed with methanol. Demethylation of P1 was performed by refluxing in a mixture of 48 wt% HBr and acetic acid for 12 h. The reaction mixture was poured into a methanol/water mixture and neutralized with aqueous ammonia. The resulting solid was stirred in 2 M hydrochloric acid (HCl) for 12 h, then filtered and thoroughly washed with water and methanol to remove residual iron species. This yielded poly(3,4‐dihydroxythiophene) (P2) in 88.6% yield, based on the initial monomer feed. In the final step, P2 was oxidized under ambient air by stirring in 33% aqueous ammonia at room temperature for 30 min, yielding P3 in nearly quantitative yield. Here, basic ammonia functions as a catalyst to promote the oxidation process similar to the aerobic oxidation of hydroquinone.^[^ ^28^ ^]^ P1 was partially soluble in chloroform, forming an emerald‐green solution (Figure 1a), whereas P2 was insoluble in common solvents. P3 was fully soluble in dimethyl sulfoxide (DMSO), yielding a bluish‐purple solution, but exhibited poor solubility in other solvents.

Size‐exclusion chromatography (SEC) analysis was performed for P3 using DMSO as eluent (Figure S2). The elution profile exhibited a bimodal distribution, where the first peak (*M*_w_ = 3.8 × 10^5^ Da, calibrated against pullulan standards) corresponds to polymer aggregates and the second peak (*M*_w_ = 2.8 × 10^3^ Da) corresponds to lower‐molecular‐weight species with minimal aggregation. Such behavior is common in rigid π‐conjugated donor–acceptor polymers, which tend to aggregate at < 100 °C, leading to apparent overestimation of molecular weights.^[^ ^29^, ^30^, ^31^ ^]^ High‐temperature SEC (∼130–200 °C) would be needed for accurate determination, but was not attempted due to the potential release of bound NH_3_ and polymer precipitation (see discussion below).

**Figure 2:**
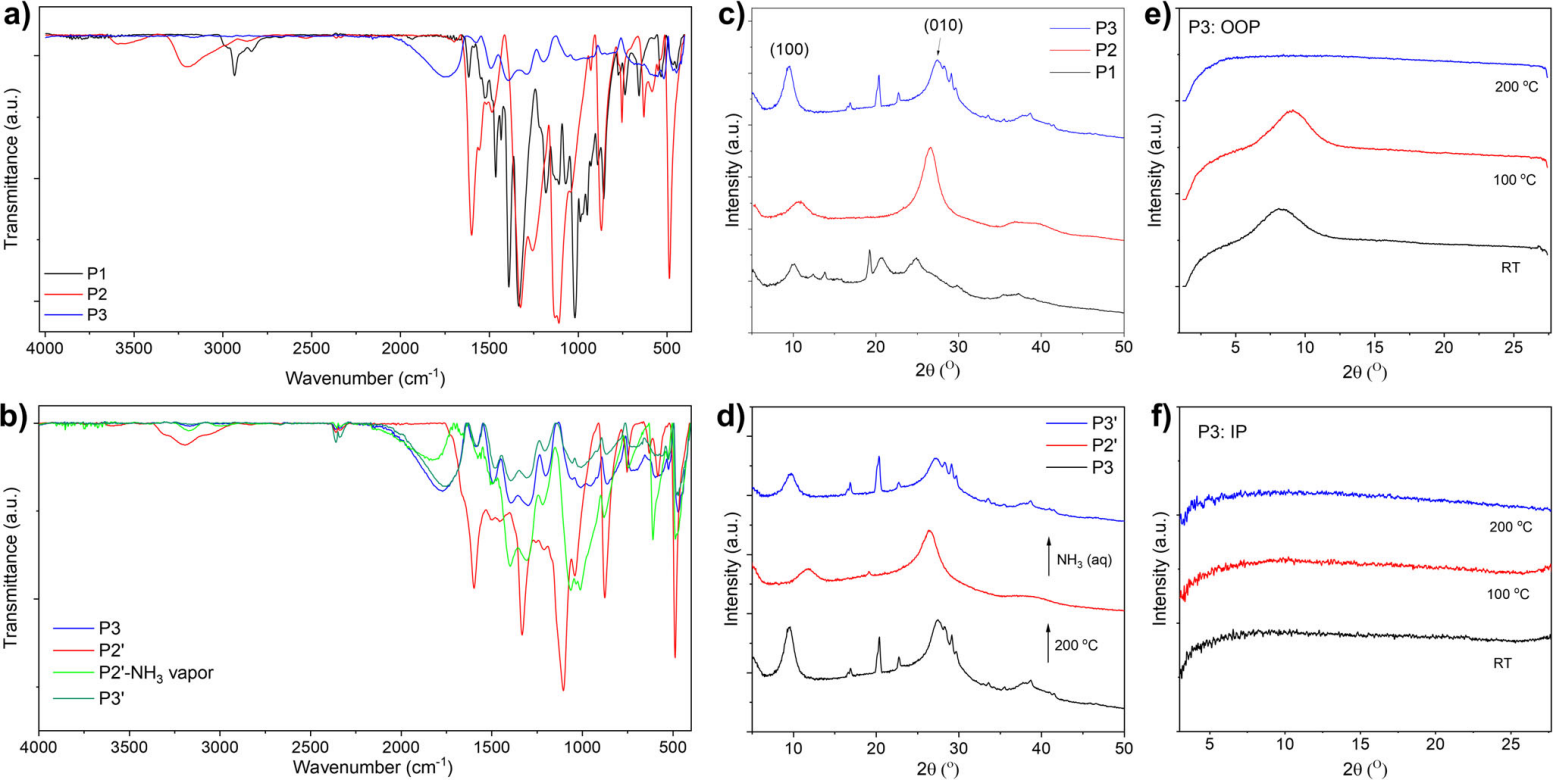
a) FTIR transmittance spectra of as‐prepared P1, P2, and P3. b) FTIR transmittance spectra of P3 before and after thermal treatment at 200 °C in air for 20 min (P2′), and after subsequent treatment with ammonia vapor (P2’‐NH_3_ vapor) or aqueous ammonia (P3′). c) XRD patterns of as‐prepared P1, P2, and P3 powders. d) XRD patterns of P3, thermally treated P2′, and regenerated P3′. e), f) 2D‐GIXD patterns of spin‐coated P3 thin films e) out‐of‐plane (OOP) and f) in‐plane (IP) orientations, as‐cast and after annealing at different temperatures.

Fourier transform infrared (FTIR) spectra were measured to analyze the chemical structures of three polymers. As shown in Figure 2a, P1 exhibited a strong peak at 2933 cm^−1^, corresponding to the methyl C─H stretching. This peak disappeared for P2 after demethylation, indicating the successful removal of methyl groups. New peaks located at 3588 and 3202 cm^−1^ are observed, which correspond to free and intermolecular bonded O─H groups,^[^ ^32^, ^33^ ^]^ indicating the formation of hydroxy groups in P2. For P3, the two O─H stretching peaks significantly weakened, while a strong peak at 1747 cm^−1^ corresponding to C═O ketone stretching was observed.^[^ ^34^ ^]^ This indicates that a portion of the dHOT units were oxidized to form the diketone dOT units by atmospheric oxygen. It was found that P3 can be converted back to P2 upon treatment with 2 M HCl, and the resulting P2 can subsequently be reconverted to P3 through exposure to ammonia, as confirmed by FTIR analysis (Figure 2b). Interestingly, heating the powdered P3 sample to 200 °C in air also resulted in the disappearance of the C═O ketone stretching band, yielding product P2′, whose FTIR spectrum closely resembles that of P2 (Figure 2b). Upon subsequent treatment of P2′ with aqueous ammonia, the obtained sample P3′ exhibited an FTIR spectrum similar to that of the pristine P3. These results suggest that the presence of ammonia is essential for stabilizing the dOT structure, as will be discussed in more detail later.

Nuclear magnetic resonance (NMR) analysis was performed to determine the structures of the two soluble polymers, P1 and P3. ^1^H NMR for P1 and P3 were performed in chloroform‐d (Figure S3) and DMSO‐d_6_ (Figure S4), respectively. P1 showed peaks located at 3.85–4.12 ppm corresponding to methoxy protons. All these signals disappeared for P3, indicating complete removal of methyl groups, which agrees with the FTIR data. Instead, a broad peak at 7.17 ppm was observed. The peak shape and chemical shift resemble the signal from a typical hydroxyl proton from phenol compounds.^[^ ^35^ ^]^ These findings suggest the presence of residual O─H groups, i.e., dHOT units, in P3 alongside the dOT unit.

To determine the dHOT/dOT ratio in P3, we added N,N‐dimethylformamide (DMF) as an internal reference, which exhibited a distinct peak at 7.95 ppm corresponding to its formyl proton, in the P3 NMR sample (Figure S6). Based on the known feed ratio and the integrated peak areas, it is determined that approximately 50% of the oxygen atoms in P3 are in the dOT form, while the other 50% remain in the dHOT form. This suggests that P3 likely adopts an alternating dHOT–dOT arrangement, as illustrated in Figure 1a. The diketone dOT units disrupt extended π‐electron delocalization along the backbone, rendering P3 a cross‐conjugated polymer, typically less efficient for charge transport than fully (linearly) conjugated analogues.^[^ ^26^ ^]^

Notably, extending the reaction time in the final synthesis step did not further increase the dOT/dHOT ratio, indicating that the dHOT–dOT structure is relatively resistant to further oxidation of the dHOT units. This behavior is reminiscent of the in situ formation of hydroquinone–benzoquinone (HQ–BQ) (also known as quinhydrone) charge‐transfer cocrystals during the oxidation of hydroquinone.^[^ ^36^ ^]^ Given that dHOT is a strong electron donor and dOT a strong acceptor, they may preferentially and perfectly form a stable intramolecular charge‐transfer complex. Attempts to further analyze the structure of P3 using ^13^C NMR were unsuccessful due to its strong aggregation tendency, resulting in poorly resolved signals (Figure S5).

Interestingly, elemental analysis (EA) of P3 reveals a relatively high nitrogen content (6.71%), corresponding to an estimated 1.5 ammonia molecules per repeat unit based on the C/N molar ratio. Given the structural similarity between the dHOT units and phenolic compounds like catechol, the hydroxyl (─OH) groups are expected to be acidic and capable of strongly coordinating with ammonia. This corresponds to an average of approximately 0.75 ammonia molecules per hydroxyl group, likely through acid–base interactions and/or hydrogen bonding, as illustrated in Figure 1.

### Crystal Structural Determination

**Figure 3:**
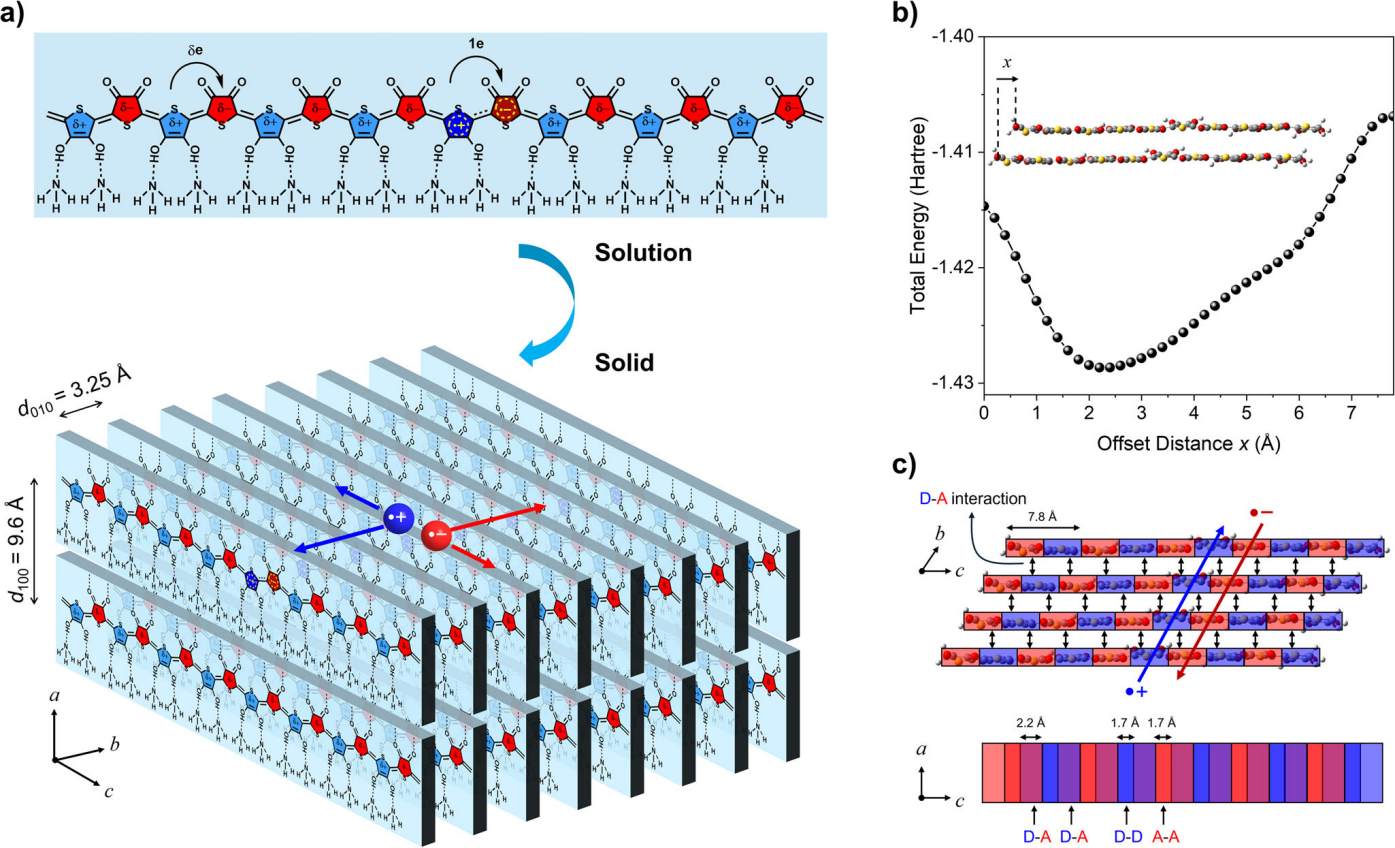
a) Proposed molecular structure of P3 in solution and its chain packing in the solid state, along with the formation of radical anions and radical cations (polarons), which act as mobile charge carriers. b) Total energy of two π–π stacked pentamer model compounds of P3 (ammonia are omitted) as a function of offset distance along the backbone direction (one repeat unit = 7.8 Å), obtained by DFT simulations.^[^ ^46^ ^]^ The minimum energy occurs at an offset of 2.2 Å (see Figure S8 for details). c) Proposed packing motif of P3 chains based on DFT results, where *a* is the interlamellar direction, *b* is the π–π stacking direction, and *c* is the backbone direction. Radical cations and anions are transported along donor and acceptor π stacks, respectively.

To investigate the chain packing structure of P3, its powder sample was analyzed using X‐ray diffraction (XRD). As shown in Figure 2c, two distinct diffraction peaks were observed at 2*θ* = 9.50° and 27.50°, corresponding to the (100) and (010) planes, respectively, which are characteristic of the lamellar and π–π stacking structures commonly found in π‐conjugated polymers (Figure 3a). The (100) peak indicates an interlamellar spacing of 9.31 Å, while the broad (010) peak centered at ∼27.5° corresponds to a π–π stacking distance of 3.25 Å, the shortest reported for a conjugated polymer so far.^[^ ^37^, ^38^, ^39^ ^]^ This unusually tight packing likely arises from strong intermolecular D–A interactions and reduced steric hindrance.^[^ ^40^, ^41^ ^]^ Notably, P3 may benefit from D–A charge‐transfer interactions akin to those in quinhydrone and other small‐molecule charge‐transfer complexes, which can exhibit π–π distances as short as 3.11 Å.^[^ ^42^, ^43^ ^]^

Direct evidence supporting this hypothesis is provided by the XRD data of the precursor polymers P1 and P2, which contain only donor units. They exhibit significantly larger π–π stacking distances of 3.58 and 3.37 Å, respectively (Figure 2c). Notably, the π–π distance of P2 is shorter than that of PEDOT (3.43 Å), suggesting reduced steric hindrance from its side groups. The interlamellar spacing also varies: P2 (8.27 Å) is shorter than P1 (8.76 Å), consistent with the smaller hydroxyl side groups in P2 compared to the bulkier methoxy groups in P1. In contrast, P3 exhibits the largest interlamellar distance (9.31 Å), attributed to the intercalation of ammonia molecules between polymer chains along the [100] direction. Additional peaks in the XRD patterns of P1 and P3 likely arise from crystalline planes involving iron salt impurities in P1 and intercalated ammonia molecules in P3, respectively.

Two‐dimensional grazing‐incidence X‐ray diffraction (2D‐GIXD) was performed on P3 thin films spin‐coated from a P3 solution in DMSO. The out‐of‐plane (OOP) diffraction pattern (Figure 2e) of the as‐cast film shows a (100) peak at 2*θ* = 8.35°, corresponding to a d‐spacing of 10.6 Å, larger than that of the powder sample, indicating an expanded interlamellar distance. No (100) peak was observed in the in‐plane (IP) direction (Figure 2f), suggesting a predominantly edge‐on orientation of the polymer backbones, which is favorable for in‐plane charge transport. Due to the angular detection limit (2θ ≤ 28.5°) of the instrument, only a partial hump above 25° was detected for the (010) π–π stacking peak.

Annealing the film at 100 °C causes the (100) peak in the OOP direction to shift to 2*θ* = 9.12° (*d* = 9.7 Å), indicating tighter lamellar packing. Further annealing at 200 °C results in the disappearance of this peak, suggesting the loss of intercalated ammonia and a transformation of P3 to P2, as confirmed by FTIR data. To validate this transformation, a P3 powder sample was heated to 200 °C and reanalyzed by XRD (Figure 2d). The thermally converted sample (P2’) showed a (010) peak at 2*θ* = 26.45° (*d* = 3.37 Å), identical to that of pristine P2 powder, while the (100) peak shifted to 2*θ* = 11.83° (*d* = 7.47 Å), indicating a more compact lamellar structure. The reduced interlamellar spacing in P2’ compared to pristine P2 suggests enhanced packing facilitated by thermal annealing.

To assess reversibility, P2’ was treated with ammonia vapor to regenerate P3 (denoted P3’). XRD analysis of P3’ revealed a π–π stacking distance of 3.27 Å, closely matching that of pristine P3, along with a slightly reduced interlamellar spacing (9.03 Å versus 9.31 Å), likely inherited from the more compact structure of P2’. These XRD results, together with FTIR data, demonstrate the excellent thermal stability of the polymer backbones and the reversible conversion between P3 and P2 through thermal annealing and ammonia treatment (Figure 1b).

To gain further insight into the role of ammonia in promoting the chain packing of P3, DFT simulations were performed on a pentamer model molecule (Figure S7 and Table S1). The results show that each hydroxyl group on a dHOT unit forms a strong hydrogen bond with an ammonia molecule, with a short N⋯H─O bond length of ∼1.70 Å. Additionally, one of the two ammonia molecules forms a second hydrogen bond (N─H⋯O─H, ∼2.02 Å) with another hydroxyl group, resulting in a double hydrogen bond that firmly anchors the ammonia molecule to the polymer backbone.

On each dOT unit, one ammonia molecule forms a double hydrogen bond using two of its hydrogen atoms: C═O⋯H─N─H⋯O═C, with each bond length around 2.24 Å. The second ammonia molecule does not directly interact with the ketone groups but forms a hydrogen bond with the first ammonia (bond length ∼2.03 Å), indicating intermolecular stabilization among ammonia molecules.

These results suggest that multiple hydrogen bonding interactions occur between ammonia and both donor and acceptor units, as well as among the ammonia molecules themselves. Compared to the dOT units, the dHOT units interact more strongly with ammonia through acid–base interactions. Notably, one ammonia molecule can form a stable double hydrogen bond with a dHOT unit, while the second may be available to bridge with a dOT on adjacent chains, helping to establish lamellar packing. Moreover, additional hydrogen bonding of ammonia molecules with hydroxyl or carbonyl groups on neighboring chains, as well as with other intercalated ammonia molecules above and below the backbone plane, may further stabilize the overall packing structure.

To further investigate the effect of intermolecular D‐A interaction on the packing motif of P3, DFT simulations of two π–π stacked pentamers were performed (Figures 3b and S8). The simulation revealed that the minimum total energy is achieved when the pentamers are offset by 2.2 Å along the backbone direction. Given that the repeat unit length is 7.8 Å (or ∼3.9 Å per thiophene unit), a significant portion of the donor unit overlaps with an acceptor unit from an adjacent chain, while a smaller portion (∼1.7 Å) forms π–π stacks with the same type of unit (Figure 3c). This donor–acceptor overlap enhances intermolecular interactions and helps achieve an ultrasmall π–π stacking distance.

Based on these findings, the proposed molecular structure and packing model of P3 are illustrated in Figure 3a. Most hydroxyl groups on dHOT units coordinate with ammonia via acid–base interactions, while hydrogen bonding occurs between the hydrogen atoms of ammonia and oxygen atoms on adjacent dOT units on the neighboring polymer chains. Along the π–π stacking direction, dHOT and dOT units form tightly packed mixed stacks stabilized by strong D–A interactions.^[^ ^43^, ^44^, ^45^ ^]^ Upon heating (e.g., at 200 °C), coordinated ammonia is released, converting P3 to P2. This transformation leads to reduced interlamellar spacing due to loss of ammonia and increased π–π stacking distance resulting from the disruption of donor–acceptor (D–A) interactions.

### Optoelectronic Properties

**Figure 4:**
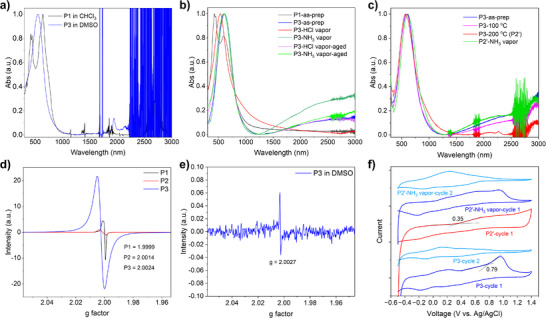
a) Normalized UV–vis–NIR absorption spectra of P1 in chloroform and P3 in DMSO. b) Normalized UV–vis–NIR absorption spectra of as‐prepared P1 and P3 films; P3 films exposed to HCl vapor (P3‐HCl vapor) and NH_3_ vapor (P3‐NH_3_ vapor); and after storing in air for 12 h (P3‐HCl vapor‐aged and P3‐NH_3_ vapor‐aged). c) Normalized UV–vis–NIR absorption spectra of P3 films as‐prepared (P3‐as‐prep), annealed at 100 °C for 20 min (P3‐100 °C) and 200 °C for 20 min (P3‐200 °C or P2′), and the P2′ film after exposure to NH_3_ vapor (P2’‐NH_3_ vapor). d) EPR spectra of P1, P2, and P3 in powder form. e) EPR spectrum of P3 in DMSO solution. f) Cyclic voltammograms of a P3 film, a P2′ film (obtained by thermal annealing of a P3 film at 200 °C), and a P2′ film after exposure to NH_3_ vapor for 10 min (P2’‐NH_3_ vapor). All films were drop‐casted onto conductive ITO substrates and used as working electrodes. Measurements were performed using an Ag/AgCl reference electrode and 1 M tetrabutylammonium hexafluorophosphate (Bu_4_NPF_6_) in anhydrous acetonitrile.

UV–vis–NIR spectroscopy measurement was conducted to investigate the optical properties of P3 in comparison with P1. In solution, P1 displays three prominent absorption peaks at 421, 448, and 641 nm, with the maximum absorption wavelength (*λ*_max_) at 641 nm, whereas P3 exhibits a single peak with a *λ*_max_ at 549 nm (Figure 4a). This result is somewhat unexpected, given that P3 adopts a cross‐conjugated configuration, unlike the linear conjugation in P1, which would typically greatly reduce the effective conjugation length.^[^ ^26^ ^]^ One possible reason is due to the strong intramolecular charge transfer between dHOT and dOT units, where the optical bandgap corresponds to the energy difference between the HOMO of dHOT and the LUMO of dOT, as observed for cross‐conjugated small molecule D–A compounds.^[^ ^47^, ^48^ ^]^

The P1 thin film exhibits three main absorption bands, centered at 425, 450, and 593 nm, with 425 nm wavelength being the *λ*_max_ (Figure 4b), consistent with spectra reported for the electrochemically synthesized counterpart.^[^ ^49^ ^]^ In contrast, P3 shows a *λ*_max_ at 600 nm in the film state, a 51 nm redshift relative to its solution spectrum, and is also redshifted compared to P1 film. These observations indicate enhanced electron delocalization in the solid–state P3, likely driven by strong intermolecular D–A interactions, namely the formation of CTCs between dHOT and dOT units (Figure 3c). The intermolecular charge transfer occurs from dHOT to dOT upon absorbing light, similar to the process occurring in small molecule CTCs.

The most striking feature of the P3 film is its ascending absorption hump in the NIR region, starting at ∼1200 nm and extending to the detection limit of the spectrometer (3000 nm). In contrast, the P1 film shows no such NIR absorption. This broad absorption is characteristic of polaronic and bipolaronic species commonly found in doped conjugated polymers.^[^ ^50^ ^]^ Given the relatively modest intensity of the NIR absorption compared to that observed in heavily doped conductive polymers, the P3 film is likely dominated by polarons rather than bipolarons, which are typically formed through the recombination of two like‐charged polarons. Compared to its film, the P3 solution shows a much weaker hump in this region, indicating a lower concentration of polarons (Figure 4a).

These observations suggest that intermolecular D–A interactions play a critical role in polaron formation. Specifically, P3 likely forms CTCs with a mixed‐stack motif between adjacent dHOT and dOT units along the π–π stacking direction, supported by the DFT simulation as illustrated in Figure 3c.

Upon exposure to HCl vapor, the P3 film exhibits a pronounced blue shift in its *λ*_max_, accompanied by a visible color change from bluish to red (Figure S9). Simultaneously, NIR absorption beyond 1200 nm diminishes. As previously discussed, HCl exposure can convert dOT units back to dHOT, as confirmed by FTIR analysis. Thus, the spectral changes suggest a reduction of dOT content in the film. Interestingly, the HCl‐treated film gradually returns to its original state after 12 h of air exposure (Figure 4b), likely due to the slow release of HCl from in situ‐formed NH_4_Cl, enabling the freed NH_3_ to re‐coordinate with dHOT units and restore the P3 structure.

In contrast, exposure to ammonia vapor led to an increase in NIR absorption, indicative of enhanced polaron formation resulting from coordination between unbound hydroxyl groups (∼25%) and NH_3_ (Figure 1b). However, this polaron peak weakens quickly, and the spectrum reverted to that of the original P3 state, suggesting that the newly absorbed NH_3_ was only weakly bound, likely interacting with dHOT units located in disordered or sterically hindered regions of the film.

Unlike P3, P1 films exhibit a pronounced red shift in *λ*_max_ upon exposure to HCl vapor, accompanied by a visible color change from yellowish green to blue (Figure S10). A new absorption feature at ∼1250 nm is also observed, characteristic of polaron species formed by HCl p‐doping. The HCl‐treated film only partially recovers to its original state after 12 h of air exposure, suggesting a stronger doping interaction between HCl and P1. In solution, HCl causes only subtle spectral changes. NH_3_ exposure has no observable effect on the absorption profile in either the film or solution state. These results indicate that P1 behaves as a typical electron‐rich p‐type conjugated polymer, distinctly different from P3, highlighting the unique behavior of P3.

Thermal annealing also affects the optical properties of P3. Annealing the P3 film to 100 °C results in a reduction of the NIR polaron absorption, while annealing at 200 °C (yielding P2′) leads to its complete disappearance (Figure 4c). A corresponding blue shift in *λ*_max_ to 569 nm is observed in the P2’ film, consistent with the conversion of P3 to P2 (Figure 1b). Notably, subsequent exposure of the P2’ film to NH_3_ vapor overnight restores the original absorption spectrum, confirming the uptake of ammonia and reformation of the P3 structure.

To confirm the presence of polarons in P3, electron paramagnetic resonance (EPR) spectroscopy was conducted. As shown in Figure 4d, the EPR spectrum of a P3 power measured at room temperature exhibits a strong, symmetric signal centered at g factor of 2.0024. Compared to polarons in other doped conjugated polymers (e.g., PEDOT:PSS (*g* = 2.0033)^[^ ^51^, ^52^ ^]^ and polyaniline (*g* = 2·0036–2·0042)^[^ ^53^ ^]^) and open‐shell diradicals (*g* = 2.0033),^[^ ^54^ ^]^ the g factor of unpaired electrons in the polarons of P3 is smaller and almost identical to that of a free electrons in vacuum (*g* = 2.0023), suggesting a higher degree of spin delocalization along a π‐conjugation system. Moreover, the g‐factor of P3 is substantially closer to the free‐electron value than those of its precursor polymers P1 and P2, which consist of donor‐only units and exhibit g‐factors of 1.9999 and 2.0014, respectively. These results collectively indicate that polarons in P3 are extensively delocalized and only weakly perturbed by their local environment, likely due to the strong charge transfer character of the polymer.

Spin quantification using TEMPO as a standard (Figure S11) reveals a radical concentration of ∼1.24 mol% relative to the thiophene units, confirming the intrinsic paramagnetic character of the polymer. In contrast, P1 and P2 show much weaker EPR signals, with radical concentrations of merely 0.044 and 0.021 mol%, respectively. These weak signals likely arise from a small population of open‐shell resonance structures common in π‐conjugated polymers^[^ ^54^, ^55^ ^]^ or slight doping by residual iron species^[^ ^56^ ^]^ in P1 and ambient oxygen in P2.

When measured in DMSO, P3 exhibits a much weaker EPR signal with a g factor of 2.0027, with a radical concentration of 0.132 mol% (Figures 4e and S12). This reduction in signal intensity aligns with the diminished polaron absorption observed in the solution‐phase UV–vis–NIR spectrum. The stark contrast between the solid and solution states underscores the critical role of the polymer's local environment in stabilizing polarons. In solution, the loss of π–π stacking, interchain donor–acceptor interactions, and hydrogen bonding via intercalated ammonia disrupts extended electronic coupling and long‐range electronic delocalization,^[^ ^57^, ^58^ ^]^ resulting in a lower population of polarons and a slight increase in the g‐factor.

Cyclic voltammetry (CV) was performed to determine the frontier molecular orbital energy levels of P3. During the first oxidative cycle, a quasi‐reversible peak appeared at 0.96 V (versus Ag/AgCl), with an onset potential of 0.79 V (Figure 4f). In the corresponding reduction scan, an irreversible process with an onset potential of −0.58 V was observed (Figure S13). Therefore, the HOMO and LUMO energy levels of P3 were determined from these onset potentials to be at −5.17 and −3.80 eV, respectively, which corresponding to a bandgap of 1.37 eV, aligned well with the optical bandgap (1.38 eV). A broad hump centered around 0.22 V was also present, likely arising from segments in which the original ammonium ions had been replaced by tetrabutylammonium (Bu_4_N^+^) from the electrolyte (see Scheme S1). Notably, in subsequent oxidative cycles, the peak at 0.96 V disappeared, leaving only the initial hump at ∼0.22 V. This behavior suggests that by the end of the first oxidation cycle, the ammonium ions had been fully replaced by Bu_4_N^+^, resulting in a modified structure more readily oxidized at lower potentials.

To further verify the reversible conversion between P3 and P2′, a P3 film deposited on a conductive ITO substrate was thermally annealed at 200 °C to generate P2′. As shown in Figure 4f, the CV profile of P2′ exhibited a distinctly different first oxidation cycle compared to that of P3, indicating significant structural transformation upon annealing. A separate P2′ sample, prepared similarly, was subsequently exposed to ammonia vapor for 10 min and subjected to CV analysis. The resulting CV curves, for both the first and second oxidative cycles, closely resembled those of the original P3, confirming that P2′ can be converted back to P3 via ammonia treatment (Figure 1b).

The HOMO energy level of P2′ was determined to be −4.73 eV, based on its onset oxidation potential (0.35 V), indicating its strong propensity for oxidation.

**Figure 5:**
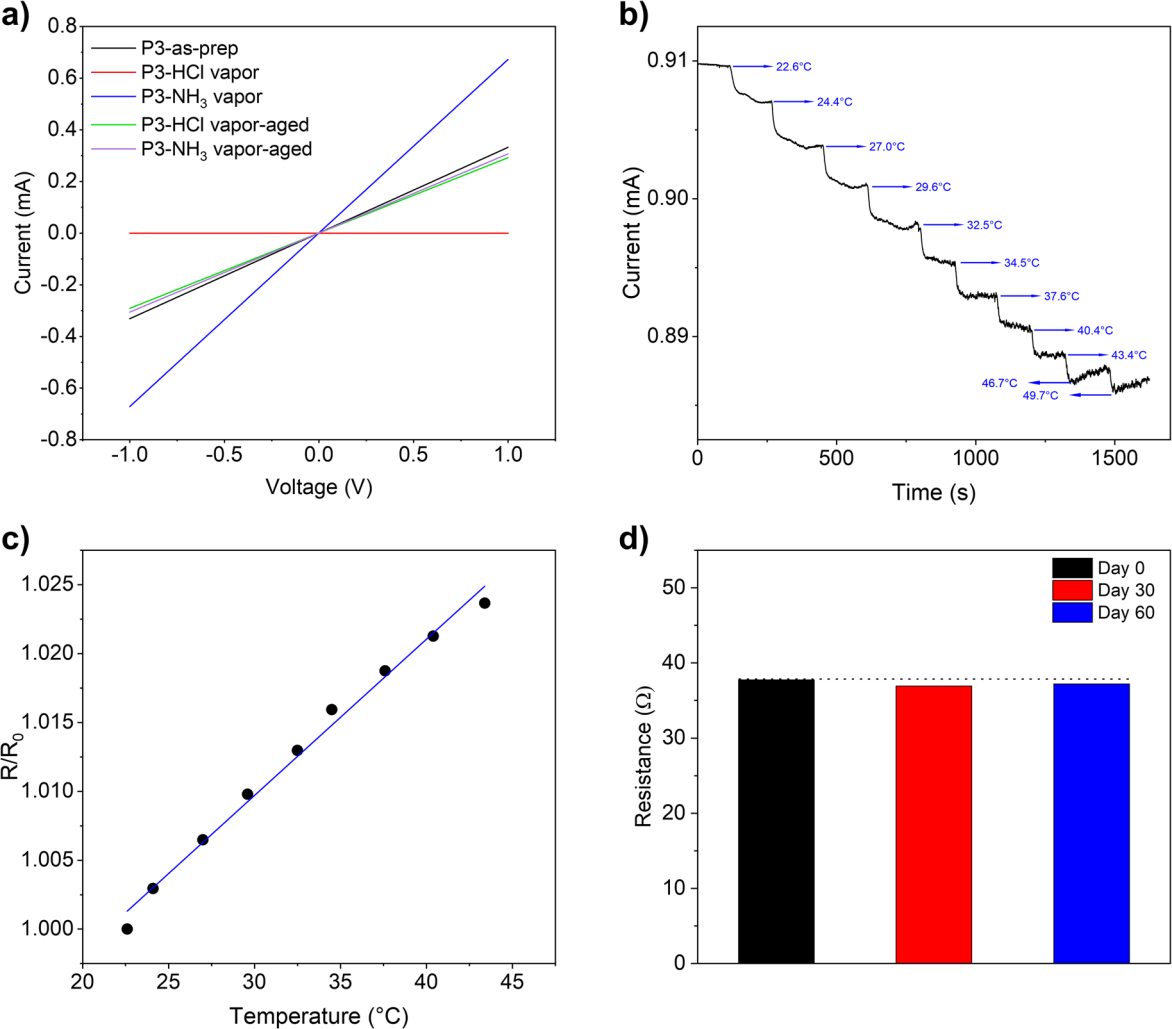
a) Current–voltage (*I–V*) characteristics of the as‐prepared P3 film; P3 film after exposure to HCl vapor (P3‐HCl vapor) for 10 min and followed by storing in air for 12 h (P3‐HCl vapor‐aged); P3 film after exposure to ammonia vapor (P3‐NH_3_ vapor) for 10 min and followed by storing in air for 12 h (P3‐NH_3_ vapor‐aged). b) Current–time response of a P3‐based resistive sensor measured at a constant voltage of 50 mV as the temperature was varied from 25 to 50 °C. c) Normalized resistance (*R*/*R*_0_) as a function of temperature in the ∼25–50 °C range, showing a linear fit with a coefficient of determination (*R* ^2^) of 0.99. d) Comparison of sensor resistance before and after storage in air for 60 days.

To evaluate the electrical conductivity of P3, a DMSO solution of the polymer was spin‐coated onto a Si/SiO_2_ wafer with interdigitated gold electrodes, forming a uniform thin film with a thickness (*t*) of ∼30–40 nm. The device featured a channel length (*L*) of 30 µm and a channel width (*W*) of 15.8 mm. A two‐terminal *I–V* measurement was performed over a voltage range of −1 to 1 V, producing a linear current–voltage response (Figure 5a), which represents an Ohmic behavior. An electrical conductivity of 0.29 ± 0.035 S cm^−1^ was measured using the four‐point probe method. Remarkably, this value is comparable to the highest reported conductivities of undoped, fully conjugated polymers and non‐conjugated radical polymers.^[^ ^17^ ^].^

Based on the molecular structure and packing of P3, the proposed charge transport mechanism is illustrated in Figure 3a,c. Strong intra‐ and intermolecular interactions between the electron‐donating dHOT units and the electron‐accepting dOT units, assisted by intercalated ammonia molecules, promote a full electron transfer in some dHOT‐dOT pairs. This results in the generation of radical cations on dHOT and radical anions on dOT, i.e., polarons, which may act as mobile charge carriers under an applied electric field and contribute to the observed electrical conduction. Donor‐only and acceptor‐only π‐stacks may provide parallel charge transport pathways, as observed in DBTTF–TCNQ,^[^ ^59^, ^60^ ^]^ while D–A π‐stacks can also serve as efficient transport channels, as demonstrated in TTF–TCNQ.^[^ ^59^, ^61^ ^]^ These π–π stacking pathways likely dominate over the cross‐conjugated backbone in contributing to the observed high mobility and conductivity.

Assuming a polymer density of 1.0 g cm^−3^, the corresponding polaron density (*N*) is calculated to be 6.54 × 10^19^ cm^−3^ based on the EPR‐measured radical concentration (1.24 mol% relative to the thiophene units). Since EPR detects both radical cations and anions, their concentrations are equal: *n*_h_ = *n*_e_ = *N*/2 = 3.27 × 10^19^ cm^−3^. The electrical conductivity (*σ*) can be expressed as in Equation 1:

(1) $$\sigma =n_{h}\;e\mu_{h}+n_{e}e\;\mu_{e}=\frac{N}{2}\;e\left(\mu_{h}+\mu_{e}\right)=Ne\mu_{avg}$$

where *e* is the elementary charge (1.602 × 10^−1^⁹ C), and µ_h_, µ_e_, and µ_avg_ are the hole, electron, and average mobilities, respectively. Using the measured conductivity and estimated *N*, the average mobility (µ_avg_) is calculated to be approximately 0.028 cm^2^ V^−1^ s^−1^. For a cross‐conjugated polymer,^[^ ^26^, ^62^ ^]^ this level of mobility is notably high and is likely facilitated by the exceptionally small π–π stacking distance, enabling efficient charge transport along the stacking direction, reminiscent of small‐molecule CTCs.

For stable hole and electron transport, the HOMO and LUMO energy levels of the polymer should be above ca. −5.6 eV and below ca. −3.7 eV, respectively.^[^ ^63^, ^64^, ^65^, ^66^ ^]^ Given P3's HOMO (−5.17 eV) and LUMO (−3.80 eV) energy levels, both radical cations and radical anions may be mobile charge carriers, contributing to the electrical conduction (Figure 3a,c).

The device was exposed to HCl and ammonia vapors under the same conditions used for the UV–vis–NIR measurements of thin‐film samples. As shown in Figure 5a, exposure to HCl vapor resulted in a complete loss of conductivity, attributed to the chemical conversion of P3 to P2 and the corresponding depletion of polarons (Figures 1b and 4b). In contrast, exposure to ammonia vapor led to a twofold increase in conductivity, consistent with the enhanced polaron population observed in the UV–vis–NIR spectrum. However, this increase was transient, as the conductivity quickly returned to its original value due to the desorption of ammonia and the subsequent loss of the newly generated polarons. On the other hand, recovery from HCl vapor exposure was significantly slower, taking approximately 12 h to return to baseline conductivity, in agreement with the UV–vis–NIR findings.

Resistor‐type temperature sensors were fabricated on flexible PET substrates with pre‐patterned interdigitated silver electrodes by blade‐coating the P3 solution in DMSO. The current of the devices was measured under a constant voltage (50 mV) at different temperatures. As shown in Figure 5b, a decrease in current was observed with increasing temperature, indicating that P3 possess a PTC sensing characteristic. A stable current level can be maintained up to around 45 °C, while further heating would cause current fluctuations.

The thermal sensitivity of the devices was evaluated by the temperature coefficient of resistance (TCR), which can be calculated using Equation 2:^[^ ^67^ ^]^

(2) $$TCR=\frac{\Delta R}{R_{0}}\times \frac{1}{\Delta T}\times 100\%$$

where *R_0_* is the electrical resistance at reference temperature *T*_0_, ∆*R* is the difference between the electrical resistance measured at temperature *T*, and ∆*T* is *T‐T*_0_.

As shown in Figure 5c, the device exhibits a nearly linear *R*/*R*_0_∼T relationship (*R* ^2^ = 0.99) over the 25–45 °C sensing range. The TCR value (the slope of the plot) is determined to be 0.113 ± 0.00045%/°C. Additionally, the same sensor device can be remeasured through multiple heating cycles with consistent TCR throughout the 60‐day testing period, demonstrating good reversibility and repeatability (Figures 5d and S14).

Resistive temperature sensors based on conductive polymers typically exhibit negative temperature coefficient (NTC) behavior, where resistivity decreases with increasing temperature,^[^ ^68^, ^69^ ^]^ primarily due to enhanced interchain charge carrier hopping at elevated temperatures.^[^ ^70^, ^71^, ^72^, ^73^ ^]^ In contrast, PTC behavior is generally observed in polymer composites incorporating conductive fillers such as metal particles, carbon nanotubes or graphene. In these systems, resistivity increases with temperature due to the thermal expansion‐induced separation of conductive fillers.^[^ ^74^ ^]^ However, PTC behavior in devices composed solely of conductive polymers is rare.^[^ ^27^ ^]^ The unusual PTC effect observed for P3 is likely related to its ultrasmall π–π stacking distance. While elevated temperatures can promote charge carrier hopping, increased molecular vibrations may disrupt the tightly packed π–π interactions, causing an expansion in stacking distances. This structural disruption impedes charge transport and can outweigh the benefits of thermally assisted hopping, leading to a net increase in resistivity with temperature.

## Conclusion

In this work, we developed poly(3,4‐dihydroxythiophene‐alt‐thiophene‐3,4‐dione) (HOT‐DOT, P3), a cross‐conjugated polythiophene that constitutes the first polymeric analogue of a quinhydrone‐like donor–acceptor charge‐transfer complex. Unlike conventional π‐conjugated polymers that rely on external dopants, P3 generates intrinsic charge carriers through its covalently fixed 1:1 donor–acceptor architecture, stabilized by ammonia coordination. This design suppresses overoxidation, enables spontaneous polaron formation, and delivers stable intrinsic conductivity.

P3 combines a narrow bandgap (1.38 eV), strong near‐infrared absorption, and notable conductivity (∼0.29 S cm^−1^), rivaling the best intrinsically conductive polymers. EPR spectroscopy confirms a high polaron concentration, while XRD reveals an ultrasmall π–π stacking distance (3.25 Å), facilitating efficient charge transport despite the cross‐conjugated backbone. DFT simulations further highlight the key role of intra‐ and interchain donor–acceptor interactions in promoting delocalization and mobility. Importantly, P3 also exhibits the rare positive temperature coefficient (PTC) effect and long‐term stability under ambient conditions. Flexible temperature sensors fabricated from P3 films demonstrate reproducible, linear responses across multiple cycles, underscoring its practical device relevance.

Overall, this work establishes polymeric charge‐transfer complexes as a powerful new design paradigm for intrinsically conductive, dopant‐free polymers, opening pathways toward stable, high‐performance materials for flexible electronics, sensors, and optoelectronic technologies.

## Supporting Information

Details on characterization methods, materials synthesis, device fabrication, and additional data on NMR, DFT, EPR, CV, and temperature sensor measurements.

## Conflict of Interests

The authors declare no conflict of interest.

## Supporting information



Supporting Information

## Data Availability

The data that support the findings of this study are available in the Supporting Information of this article.
