# Supplementary material for: An Intrinsically Conductive Cross‐Conjugated Polymer with a Quinhydrone‐Like Donor–Acceptor Charge‐Transfer Network
Source: Angew Chem Int Ed Engl. 2025 Nov 4;64(52):e18109. doi: 10.1002/anie.202518109 (PMC12723467; doi:10.1002/anie.202518109)
Supplement: Supplementary file 1 — Supporting Information [file ANIE-64-e18109-s001.pdf]

Copyright WILEY-VCH Verlag GmbH & Co. KGaA, 69469 Weinheim, Germany, 2018.

Supporting Information

An Intrinsically Conductive Cross-Conjugated Polymer with a Quinhydrone-Like Donor–Acceptor Charge-Transfer Network

Naixin Zhao, Yonglin Wang, Xin Jin, and Yuning Li*

N. Zhao, Y. Wang, X. Jin, Prof. Y. Li

Department of Chemical Engineering

Waterloo Institute for Nanotechnology (WIN)

University of Waterloo

200 University Ave West, Waterloo, Ontario N2L 3G1, Canada

E-mail: yuning.li@uwaterloo.ca

*Corresponding author

1. Materials

3,4-Dimethoxythiophene, iron (III) chloride, 48 wt% hydrobromic acid, and 33 wt% ammonia hydroxide were purchased from commercial sources (Aldrich, TCI, VWR, and Oakwood) and used without further purification, unless stated otherwise. Anhydrous solvents were purchased from Sigma-Aldrich and used as received. A heavily n-doped Si wafer (4-inch) with a 300 nm polished SiO_2 layer was purchased from University Wafer. Chromium rods and gold ingots (99.9%) used for thermal evaporation were purchased from Angstrom Engineering.

2. Characterizations

2.1 Instruments

Nuclear magnetic resonance (NMR) spectra were obtained using a Bruker DPX 300 MHz spectrometer. UV-Vis NIR spectra were obtained using a Cary 7000 UMS UV-Vis-NIR spectrophotometer. Cyclic voltammetry (CV) measurements were performed using an indium-doped tin oxide (ITO)-coated glass working electrode, an Ag/AgCl reference electrode, and a Pt disk counter electrode in a 0.1 M tetrabutylammonium hexafluorophosphate solution in anhydrous acetonitrile at a scan rate of 20 mV/s. Ferrocene was used as the reference, which has a HOMO energy level of -4.8 eV.[1] The HOMO energy level of the polymer was calculated using the equation of EHOMO (eV) = $-(E_{ox,onset} – E_{Fc/Fc+,onset})$ – 4.8 eV, where $E_{Fc/Fc+,onset}$ was determined by scanning at same scan rate with blank electrodes plus 50 mg of ferrocene added to the electrolyte (Figure S11a). The resulting CV diagram has an oxidative onset voltage of 0.42 V corresponding to a $E_{Fc/Fc+,onset}$ = 0.42 eV. X-ray diffraction (XRD) measurements were performed on a Bruker D8 Advance diffractometer with Cu Ka radiation (λ = 0.15406 nm) using polymer powders or polymer films spin-coated on a silicon wafer. Electrical conductivity measurements were performed using an Ossila Xtralien X100 four-probe source measure unit. Temperature sensor and two-terminal *I*–*V* measurements were performed using an Agilent B2912A Semiconductor Analyzer. Electron paramagnetic resonance (EPR) spectroscopy was measured on a Bruker EMXmicro PremiumX spectrometer. Size exclusion chromatography (SCE) analysis was performed by PolyAnalytik Inc. using Viscotek TDA302 system coupled with a GPCmax instrument. The analysis was carried out under 60 °C using DMSO as the eluent and pullulan as the calibration standard. Computer

simulations were carried out by Gaussian 16 software using the density functional theory (DFT), as approximated by the B3LYP functional, and using the 6-31G (d, p) basis set. For interchain stacking simulations, PM6D3 basis set was used, and Gaussian input files were generated using a custom Python script and Molclus program.[2]

2.2 Device Fabrication

Gold electrode pairs with with a channel length of 30 μm and a channel width of 15.8 mm were patterned on an Si/SiO_2 wafer with a 300 nm-thick SiO_2 layer using a conventional photolithography process followed by thermal deposition of chromium and then gold. For conductivity measurements, the substrates were cleaned by submerging and sonicating them in deionized water for 20 minutes. The process was repeated with acetone and isopropanol. The cleaned substrates were then dried with compressed nitrogen and treated with oxygen plasma under low air flow for 2 minutes. P3 thin films were deposited onto the substrates by spin-coated (3000 rpm) 50 µL of polymer solution (10 mg mL^{-1} in DMSO) for 300 seconds, yielding a thin film with a thickness of approximately 30-50 nm. Prior to the fabrication, the devices were preheated at 100 °C to ensure solvent evaporation.

For temperature sensing experiments, PET substrates with patterned interdigitated Ag electrodes were used. The substrates were obtained from the Institute for Graphic Communications and Printability (ICI), Montreal, Canada. Ag electrodes were printed by an industrial roll-to-roll press with flexography printing units (OMET Varyflex V2) at a speed of 15 m/min using a water-based nano-silver ink from NovaCentrix (PFI600) with adjusted viscosity and drying retardant.[3] The printed devices were dried in-line with hot air at 100 °C. The produced Ag electrodes (~350 nm thick) have a conductivity of 4.40×10^4 S cm^{-1}.

The PET substrates with interdigitated printed silver electrodes with a W/L ratio of 10000 were cleaned by washing with isopropanol and acetone. The substrates were dried using compressed nitrogen before blade-coating the P3 polymer solution in DMSO with a concentration of 10 mg mL^{-1} onto the cleaned substrate. The devices were then stored in a vacuum chamber for 12 hr to remove the excess solvent.

3. Synthesis

*3.1 Synthesis of Poly(3,4-dimethoxythiophene) (**P1**)*

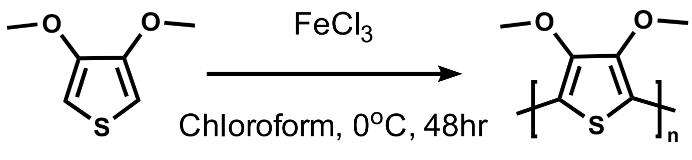

M1 **P1**

To a two-necked 100 mL round-bottom flask, iron (III) chloride (2.25 g, 13.87 mmol) was added. The system was purged with N_2 before anhydrous chloroform (20 mL) was syringe-injected through the rubber septum to suspend the mixture. The suspension was sonicated to make a homogeneous dispersion of the iron (III) chloride before chilling to 0 °C with constant stirring. Then, 3,4-dimethoxythiophene (M1) (0.50 g, 3.47 mmol) pre-dissolved in 5 mL anhydrous chloroform was syringe-injected to the suspension dropwise. The mixture was kept under stirring at 0 °C for 48 hr. Upon completion, the solvent was removed under reduced pressure. The black solid was washed with methanol to remove excess iron (III) chloride, then vacuum filtrated to obtain P1 as a black solid. Yield: 0.59 g, which exceeds the theoretical yield (0.49 g) due to the trapping of iron species, which could be removed in the next step. Elemental analysis: C: 46.34%, H: 3.42%.

*3.2 Synthesis of Poly(3,4-dihydroxythiophene) (**P2**)*

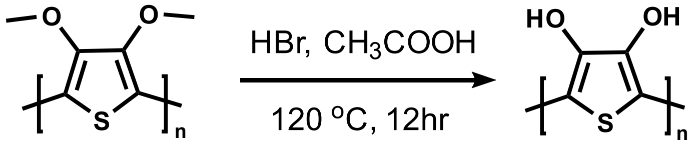

P1 **P2**

To a one-necked 100 mL round-bottom flask, P1 from the previous step was added quantitatively. Then, 48 wt% hydrobromic acid (12 mL) and glacial acetic acid (6 mL) were added. The system is then heated with stirring under reflux for 12 hr in ambient conditions. Upon completion, the polymer suspension was poured in a 4:1 methanol: deionized water (v/v) solvent and neutralized to pH = 7 with 33 wt% ammonium hydroxides. The crude product was obtained through vacuum filtration, then re-acidified by stirring the polymer suspension in a 2M HCl aqueous solution for

12 hr under room temperature. Finally, the product P2 was collected with another vacuum filtration, obtained as a black solid (351 mg, 88.6%). Elemental analysis: C: 36.12%, H: 1.58%.

3.3 Synthesis of Poly(3,4-dihydroxythiophene)-thiophene-3,4-dione ***(HOT-DOT, P3)***

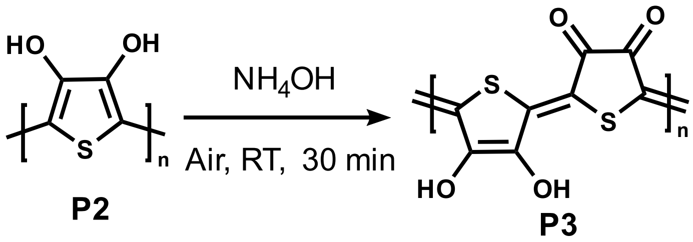

To a one-necked 100 mL round-bottom flask, 30 mL of 33 wt% ammonium hydroxide was added with stirring in the air. Then, P2 (100 mg) was poured into the solution and stirred for 30 minutes. Upon completion, the reaction was quenched by pouring it into a 4:1 methanol: deionized water (v/v) solvent, then vacuum filtrated and dried to obtain P3 as a black solid in quantitative yield (100 mg, ~100%). Elemental analysis: C: 30.24%, H: 2.51%, N: 6.71%.

4. Additional data

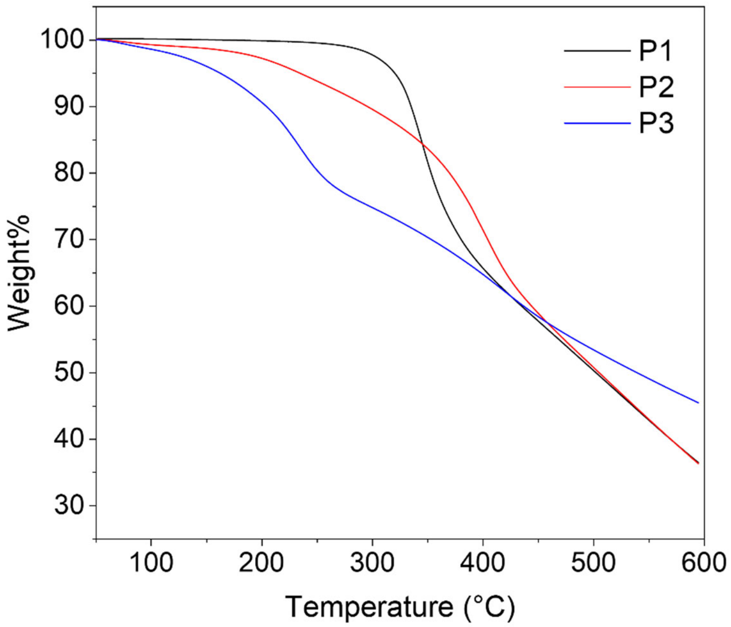

Figure S1. Thermalgravimetric analysis (TGA) curves of P1, P2, and P3 measured at a heating rate of 10 °C min^{-1} under N_2.

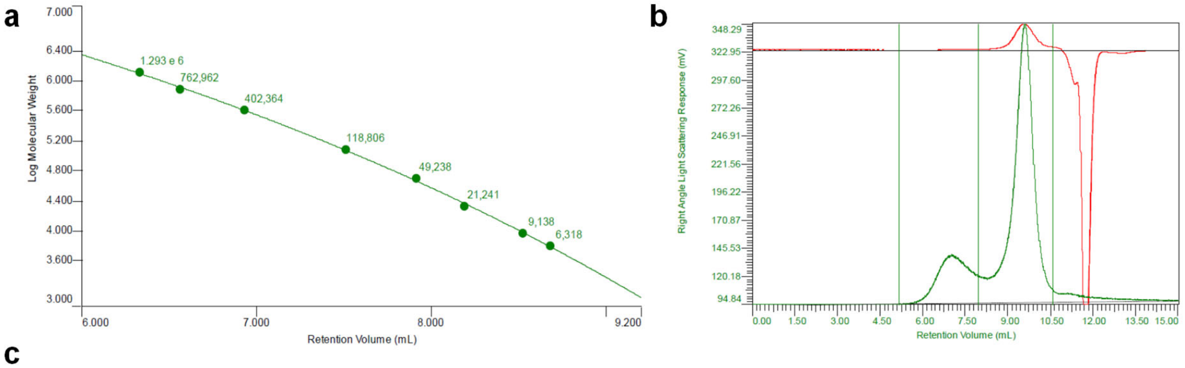

c

Sample Code	Peak	V_p (mL)	M_n (Da)	M_w (Da)	M_z (Da)	M_p (Da)	M_w/M_n (PDI)
PA-SMPL1002847	1	7.63	223,421	389,150	660,543	167,063	1.742
	2[a]	9.55	409	2,764	17,468	1,198	6.755

Figure S2. SEC data of P3 measured at 60 °C using DMSO as the eluent. (a) Pullulan calibration curve (third-order fit) obtained from refractive index (RI) detection in SEC measurements performed in 0.1 M LiBr/DMSO at a flow rate of 0.5 mL min^{-1} and 60 °C; (b) Elution profile of P3 detected by RI (red) and right-angle light scattering (RALS, green); and (c) Summary of SEC analysis results for P3.

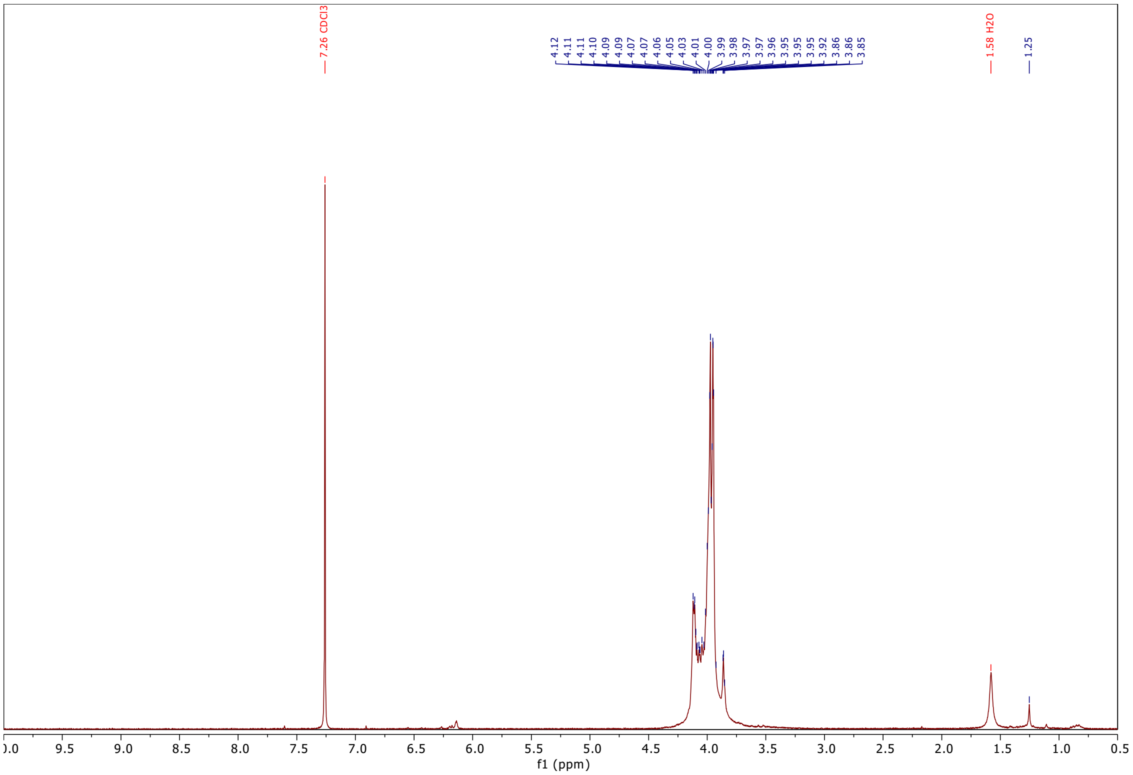

Figure S3. 300 MHz ^{1}H NMR spectrum of P1 in chloroform-*d*.

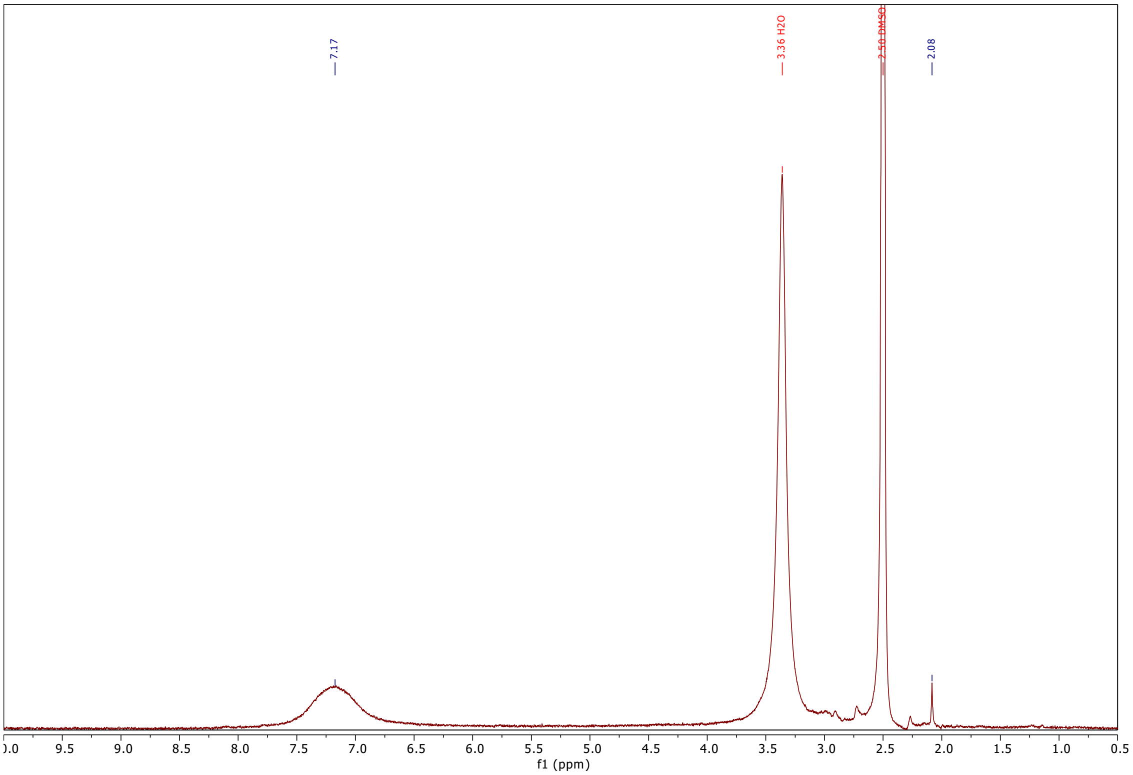

Figure S4. 300 MHz ^{1}H NMR spectrum of P3 in DMSO-d_6.

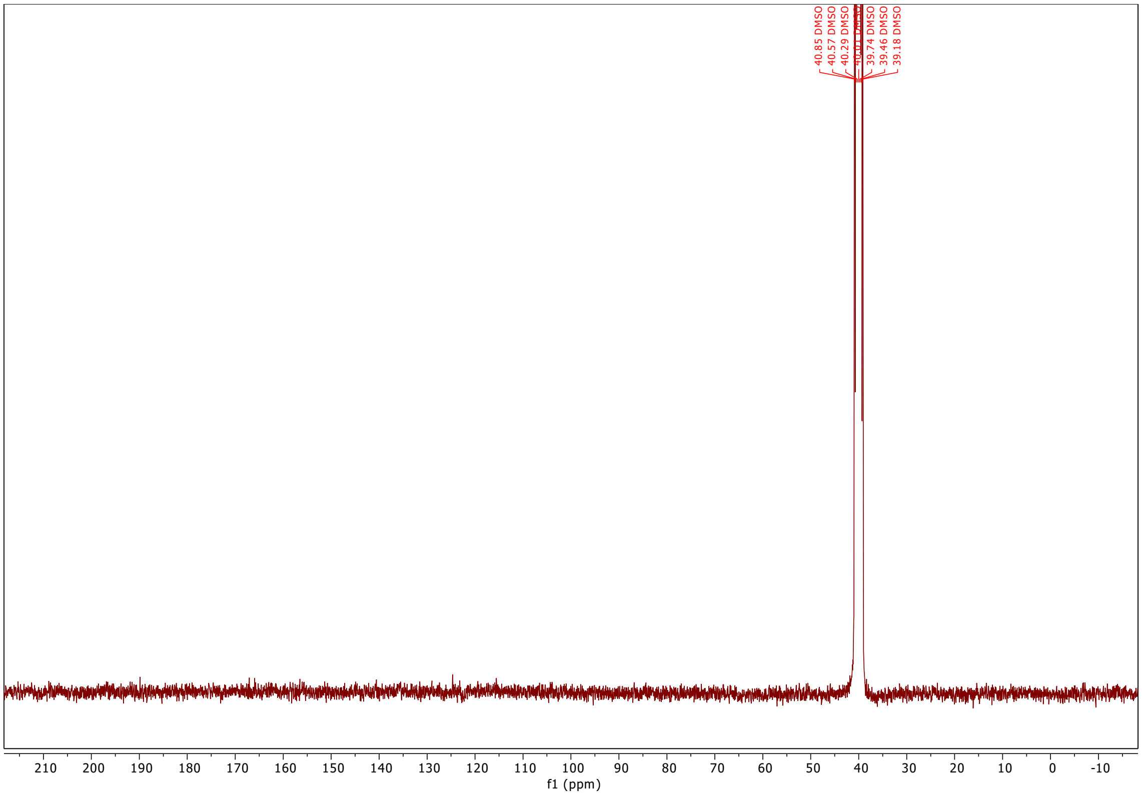

Figure S5. 300 MHz ^{13}C NMR spectrum of P3 in DMSO-d_6

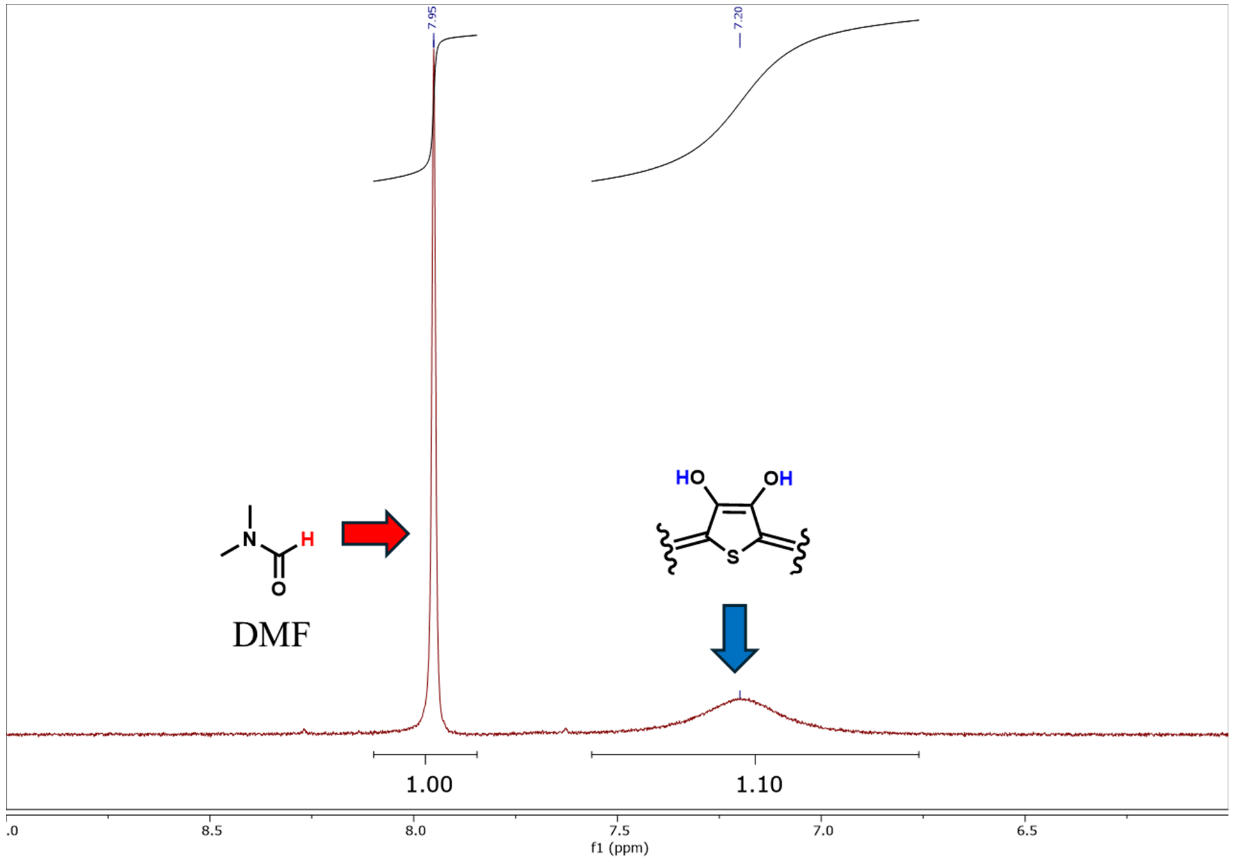

Figure S6. 300 MHz ^{1}H NMR spectrum of P3 with DMF standard in DMSO-d_6. The molar ratio between DMF and P3 was 1:1.1, resulting in a proton integration between the formaldehyde proton and hydroxy proton ratio of 1:1.1 (1:2.2 if 100% hydroxy). The hydroxy abundance of P3 is estimated to be 50%.

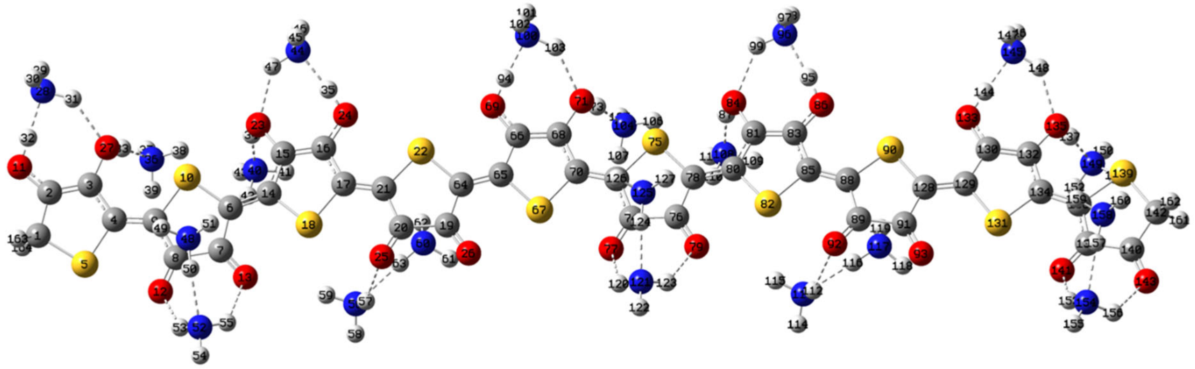

Figure S7. Energy-minimized geometry of a P3 pentamer model with two ammonia molecules coordinated to each dHOT or dOT unit, obtained via DFT simulation. Dashed lines indicate possible hydrogen bonds; corresponding bond lengths are listed in Table S1.

Table S1. Hydrogen bond lengths in the P3 pentamer model shown in Figure S6, with two ammonia molecules coordinated to each dHOT or dOT unit, as determined by DFT simulation.

	N···H–O, Å	N-H···O–H, Å	N–H···O=C, Å	N–H···N–H, Å
N28-H32	1.658			
N36-H33	1.719			
N40-H34	1.837			
N44-H35	1.650			
N100-H94	1.651			
N104-H73	1.706			
N108-H87	1.834			
N96-H95	1.646			
N145-H144	1.642			
N149-H137	1.679			
H31-O27		2.012		
H47-O23		2.044		
H103-O71		2.023		
H99-O84		2.050		

H148-O135		2.020		
H53-O12			2.191	
H55-O13			2.301	
H57-O25			2.157	
H120-O77			2.282	
H123-O79			2.246	
H112-O92			2.166	
H153-O141			2.150	
H156-O143			2.436	
H50-N52				2.018
H63-N56				2.059
H124-N121				1.998
H116-N113				2.051
H157-N154				2.001
**Average**	**1.702±0.075**	**2.030±0.016**	**2.241±0.098**	**2.025±0.028**

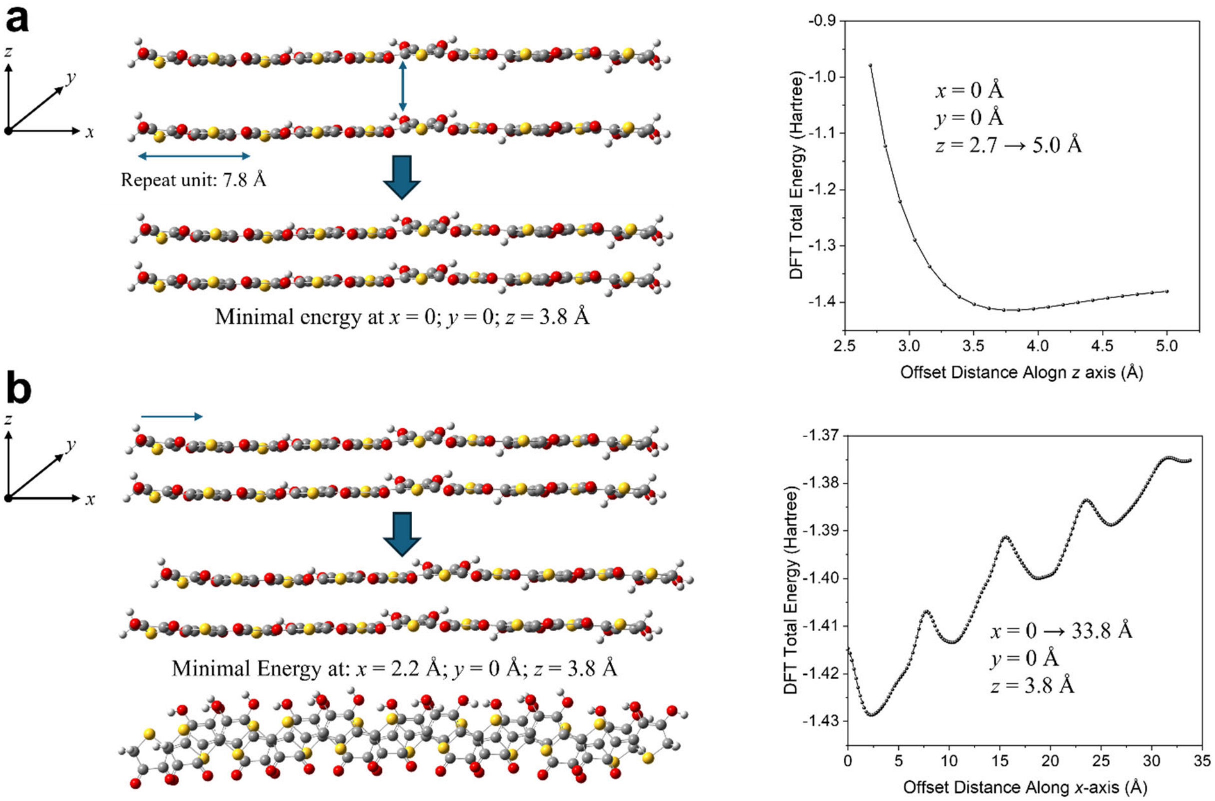

Figure S8. DFT simulation of interchain π–π stacking between two P3 pentamer model molecules. (a) Total energy as a function of interchain distance along the *z*-axis with fixed lateral offsets at ($x = 0$, $y = 0$), showing a minimum at $z = 3.8$ Å. (b) Further optimization by varying the offset along the *x*-axis reveals a lower energy minimum at $z = 2.2$ Å. Each pentamer was individually energy-minimized using DFT prior to the stacking simulation. The length of one repeat unit (dHOT–dOT) is approximately 7.8 Å.

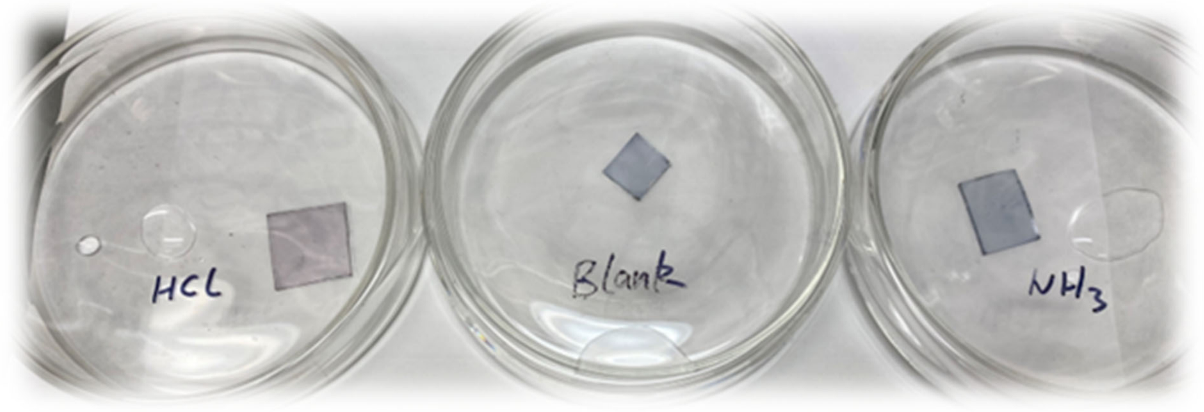

Figure S9. Photographs of the as-prepared P3 film, after exposure to HCl vapor, and after exposure to ammonia vapor for 10 min, showing different colours.

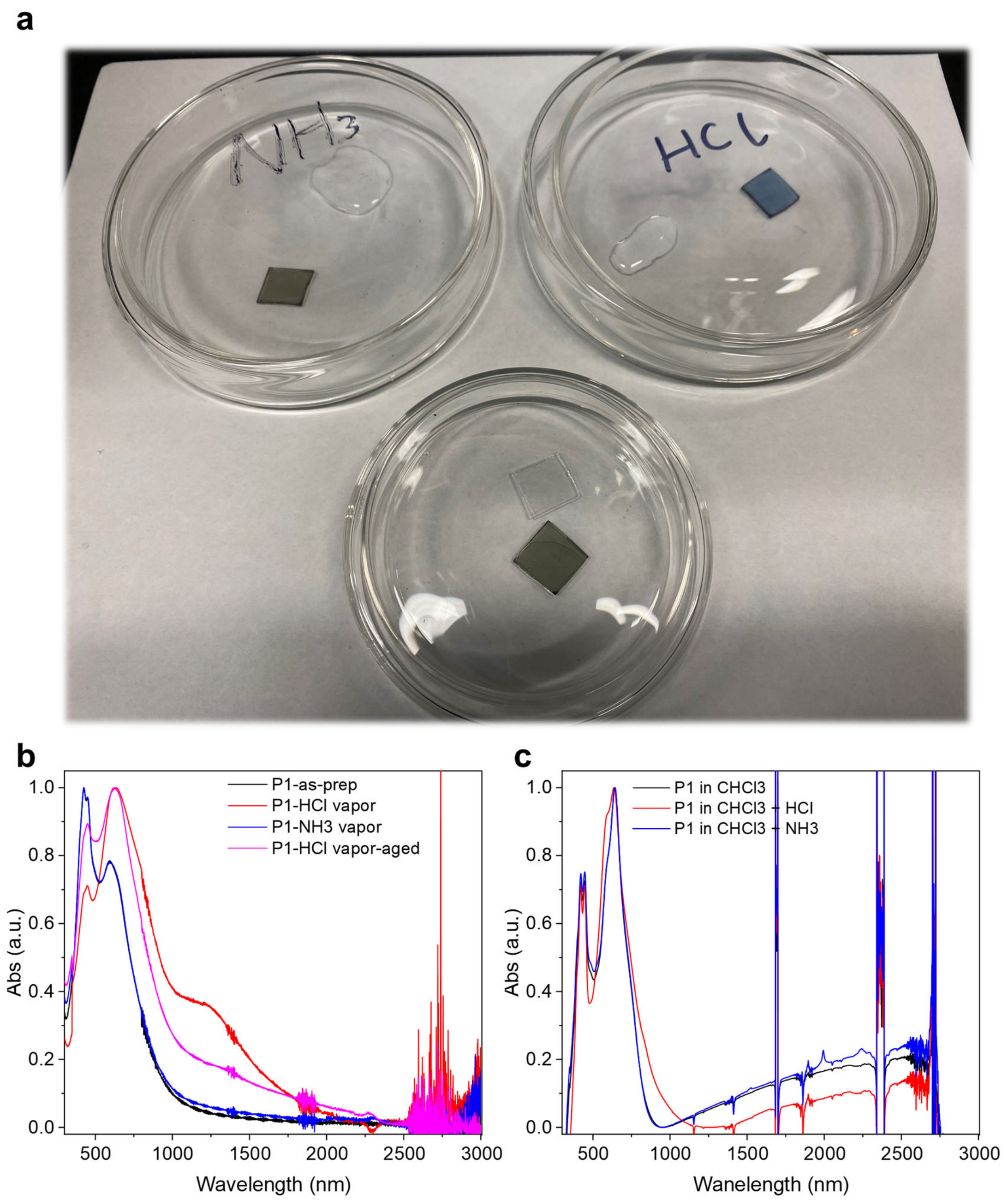

Figure S10. (a) Photographs of the as-prepared P1 film (bottom), after exposure to HCl vapor (top right), and after exposure to NH_3 vapor (top left) for 10 min. (b) Normalized UV–Vis–NIR absorption spectra of the as-prepared P1 film, P1 film exposed to HCl vapor (P1-HCl vapor) and after 12 h in air (P1-HCl vapor-aged), and P1 film exposed to NH_3 vapor (P1-NH_3 vapor). (c) Normalized UV–Vis–NIR absorption spectra of P1 in chloroform, P1 solution (3.5×10^{-7} mol repeat units) with 5 μL (~6×10^{-5} mol HCl) of 37 wt% HCl solution (P1 in $CHCl_3$ + HCl), and P1 solution with 5 μL (~9×10^{-5} mol NH_3) of 33 wt% NH_4OH solution.

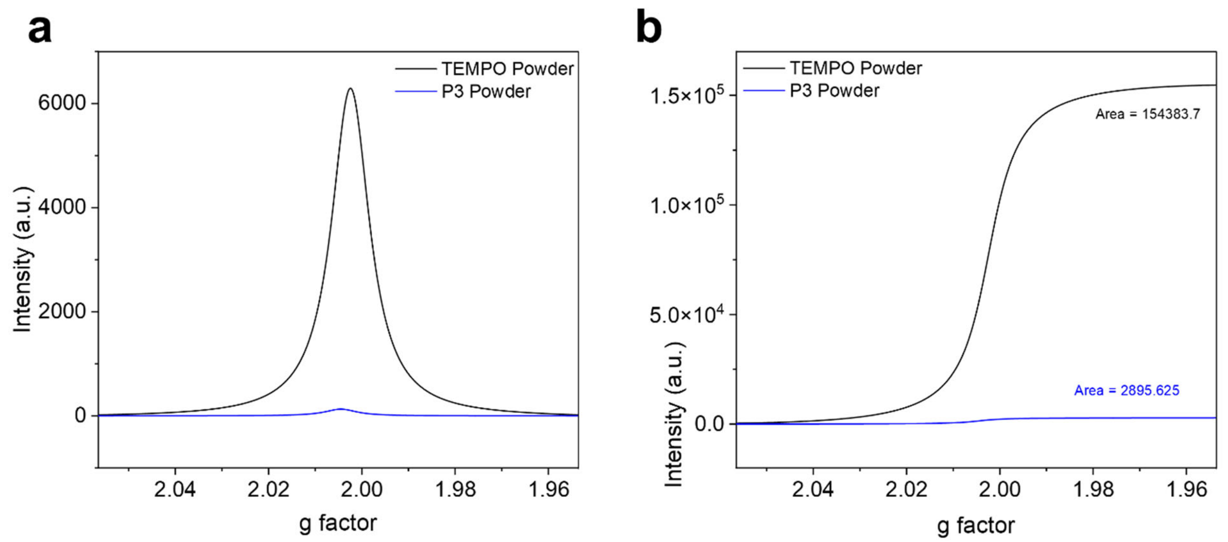

Figure S11. (a) First integration and (b) second integration of EPR spectra for TEMPO standard (0.00813 mmol) and P3 (0.0123 mmol thiophene units) in powder form. The radical content of TEMPO equals its sample amount (1:1 molar ratio), while the radical content of P3 (0.000152 mmol) was determined by applying the ratio of the second integrals. Based on this, the radical abundance in P3 is estimated to be 1.24 mol%.

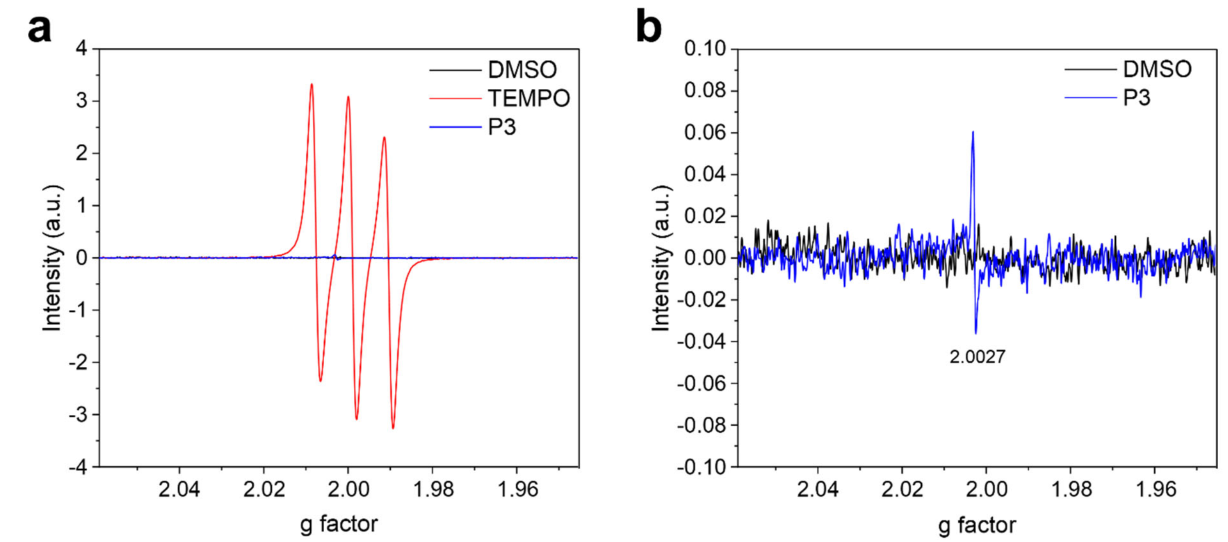

Figure S12. EPR spectra of TEMPO and P3 in DMSO solutions (5 mg mL^{-1}), along with a DMSO-only control.

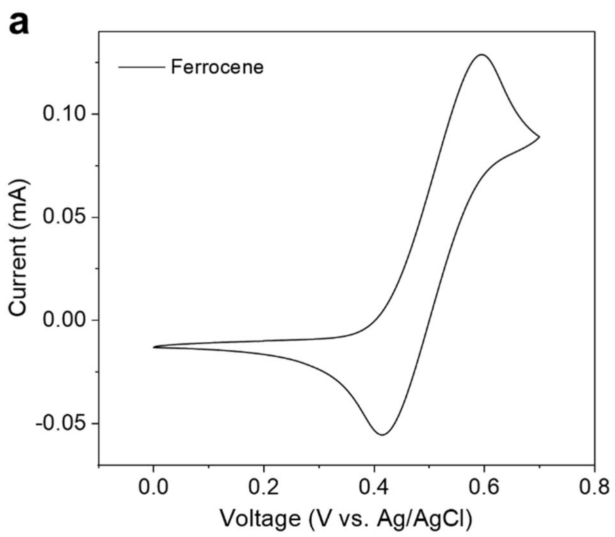

Figure S13. Cyclic voltammograms of (a) ferrocene in solution and (b) a P3 film drop-casted onto a conductive ITO measured using an Ag/AgCl reference electrode and 1 M tetrabutylammonium hexafluorophosphate (Bu_4NPF_6) in anhydrous acetonitrile at a scan rate of 20 mV s^{-1}.

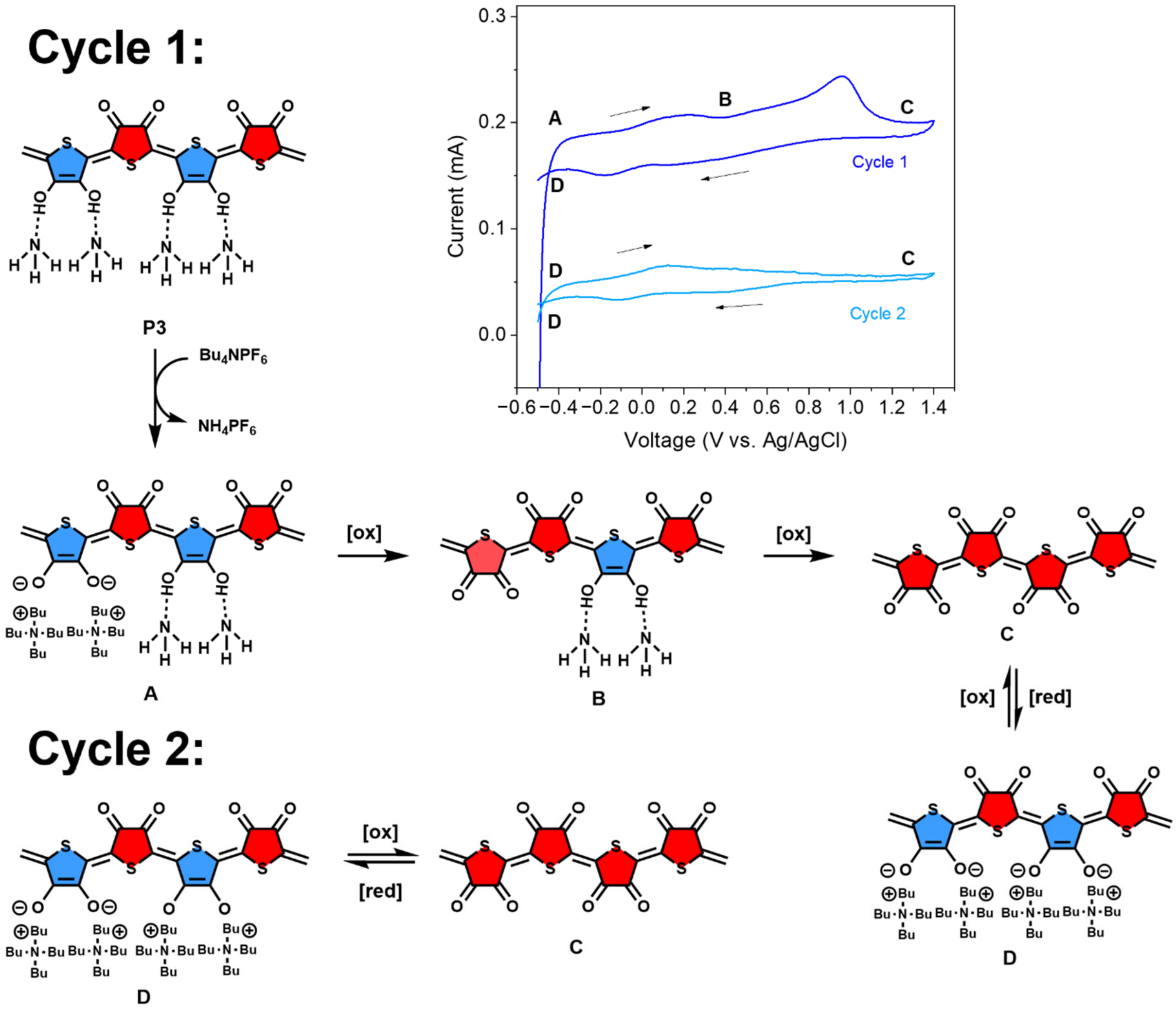

Scheme S1. Proposed replacement of ammonium (NH_4^+) in P3 by tetrabutylammonium (Bu_4N^+) from the electrolyte, along with the associated electrochemical reactions occurring during the first and second oxidative cycles. The inset shows the cyclic voltammograms of P3 during the first and second oxidative cycles (corresponding to those shown in Figure 4f).

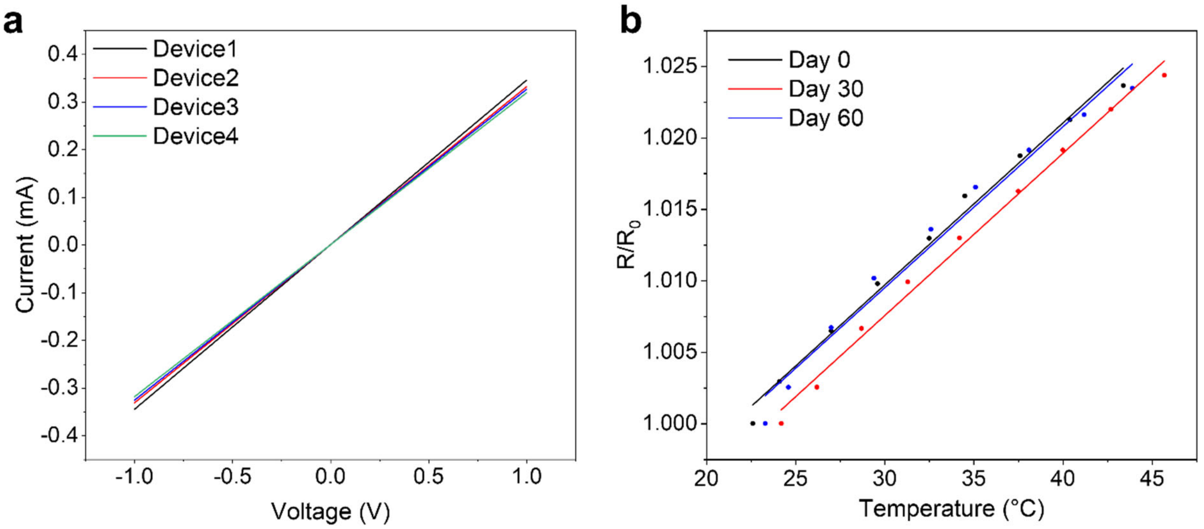

Figure S14. (a) Current–voltage (I–V) characteristics of four devices with spin-coated P3 thin films, normalized by film thickness. (b) Temperature-dependent normalized resistance (R/R_0) in the ~25–50 °C sensing range, showing linear fits for three separate cycles measured over a 60-day testing period.

5. Reference

[1] J. Pommerehne, H. Vestweber, W. Guss, R. F. Mahrt, H. Bässler, M. Porsch, J. Daub, "Efficient two layer leds on a polymer blend basis," *Adv. Mater.* **1995**, *7*, 551–554.

[2] Lu, T. Molclus Program, Version 1.12. http://www.keinsci.com/research/molclus.html (accessed 2025-07-04).

[3] M. Zhuldybina, L.-P. Beliveau, M. Mansourian, X. Ropagnol, N. D. Trinh, C. Bois, F. Blanchard, in *2021 46th Int. Conf. Infrared Millim. Terahertz Waves IRMMW-THz*, IEEE, Chengdu, China, **2021**, pp. 1–2.
